# Transcription dosage compensation does not occur in Down syndrome

**DOI:** 10.1186/s12915-023-01700-4

**Published:** 2023-11-10

**Authors:** Samuel Hunter, Jo Hendrix, Justin Freeman, Robin D. Dowell, Mary A. Allen

**Affiliations:** 1 Molecular, Cellular, and Developmental Biology, University of Colorado Boulder, Boulder, 80301 USA; 2 BioFrontiers Institute, University of Colorado, Boulder, 80309 USA; 3 Computational Bioscience, The University of Colorado Anschutz Medical Campus, Aurora, CO USA; 4 Center for Genes, Environment and Health, National Jewish Health, Denver, CO USA; 5 Linda Crnic Institute for Down Syndrome, 80045 Aurora, USA; 6 Crnic Boulder Branch, BioFrontiers, Boulder, 80309 USA

**Keywords:** Down syndrome, GRO-seq, RNA-seq, DNA-seq, Dosage compensation

## Abstract

**Background:**

The increase in DNA copy number in Down syndrome (DS; caused by trisomy 21) has led to the DNA dosage hypothesis, which posits that the level of gene expression is proportional to the gene’s DNA copy number. Yet many reports have suggested that a proportion of chromosome 21 genes are dosage compensated back towards typical expression levels (1.0×). In contrast, other reports suggest that dosage compensation is not a common mechanism of gene regulation in trisomy 21, providing support to the DNA dosage hypothesis.

**Results:**

In our work, we use both simulated and real data to dissect the elements of differential expression analysis that can lead to the appearance of dosage compensation, even when compensation is demonstrably absent. Using lymphoblastoid cell lines derived from a family with an individual with Down syndrome, we demonstrate that dosage compensation is nearly absent at both nascent transcription (GRO-seq) and steady-state RNA (RNA-seq) levels. Furthermore, we link the limited apparent dosage compensation to expected allelic variation in transcription levels.

**Conclusions:**

Transcription dosage compensation does not occur in Down syndrome. Simulated data containing no dosage compensation can appear to have dosage compensation when analyzed via standard methods. Moreover, some chromosome 21 genes that appear to be dosage compensated are consistent with allele specific expression.

**Supplementary information:**

The online version contains supplementary material available at 10.1186/s12915-023-01700-4.

## Background

Trisomy 21 (T21, the major cause of Down syndrome) is the most prevalent aneuploidy in the human population [1]. Individuals with Down syndrome have stereotypical physical features and some degree of intellectual disability. In addition, they have an increased risk for some health problems, such as congenital heart disease, and a decreased risk of others, including solid tumor formation (see review [2]). This altered risk profile arises primarily from the effect of higher levels of transcription of genes encoded on chromosome 21 [3–5]. The increase in both DNA copy number and transcription has led to the DNA dosage hypothesis: gene transcription levels are proportional to DNA dosage [6, 7].

Dosage compensation is any mechanism that modulates gene expression to compensate for increased DNA dosage [8]. The most famous and well-studied dosage compensation mechanism involves X inactivation to balance sex chromosome expression levels [9]. In contrast, no dosage compensation mechanism is known to exist for an entire mammalian autosomal chromosome [8]. In Down syndrome, an autosomal chromosome is triplicated, thus, there is tremendous interest in whether dosage compensation exists for any genes on chromosome 21.

Numerous studies of gene expression in Down syndrome cells have reported gene expression levels that do not strictly follow DNA dosage [10–15]. Others have contradicted these findings, arguing instead that most genes follow the expected 1.5-fold increase, with only a few genes showing lower-than-expected expression levels [3, 5]. In part, the differences in opinion arise from how each study defines dosage compensation. For example, a permissive definition regards every gene below the expected DNA dosage (1.5 median fold change) as dosage compensated [15], but this ignores normal, inherent statistical variation. A more principled approach looks for deviations from the 1.5 median fold change that exceed statistical expectation [5]. Regardless of the methodology used for identifying dosage compensation, all studies identify at least a small number of genes expressed below expectation, suggesting they may be dosage compensated.

Here we sought to identify the sources of apparent dosage compensation, both technical and molecular. Thus we first focus on short read sequencing data and the statistical methods of assessing differential expression. Differential expression tools, such as DESeq2, provide a systematic, reproducible method of identifying true differences while minimizing false positives, typically by accounting for the underlying noise inherent to these data [16]. These tools are exceptionally well designed to discover differential gene expressions when the data fit the expectation of the tool (see review [17]).

Typically when employed to analyze trisomy data, it is assumed the tools will work well without modification. Yet to date, no study has examined how the presence of an extra copy of chromosome 21 impacts the typical differential expression analysis pipeline. Therefore, we first dissect the typical analysis pipeline to identify issues that could lead to erroneous identification of dosage compensation. To this end, we simulated transcription data sets for both a disomic (D21) and trisomic individual where no dosage compensation is present by design (i.e., all chromosome 21 genes measure at the expected 1.5× change). Using the simulated data, we adjust the analysis pipeline to accurately account for the trisomy nature of the data. We then apply our trisomy-aware analysis pipeline to both steady-state RNA-seq and nascent transcription data, generated from a family where one child has Down syndrome. Our study finds that only a few chromosome 21 genes are expressed or transcribed at lower than the 1.5× expectation.

We hypothesize that the few genes with lower than expected levels may reflect individual allele effects. Research suggests inter-individual variation is a more substantial contributor to differential expression in T21 studies than sex or aneuploidy status [3, 5]. Consistent with this, tremendous variability in expression levels exists within the population of typical, diploid humans [18]. For example, large-scale studies of gene expression among typical humans find that 83% of genes are differentially expressed between subsets of individuals [19]. Expression quantitative trait loci (eQTL) studies seek to identify loci genome-wide that contribute to observed variation in gene expression levels [20, 21]. Thus we DNA sequence the family to trace allelic inheritance and find that most of the genes with lower-than-expected transcription arise from genetic variations that lead to lower expression levels (known eQTLs).

Our findings show no dosage compensation at either transcription or steady-state RNA levels. RNA-seq analysis pipelines, when not trisomy aware, can lead to the appearance of dosage compensation when there is none. Furthermore, our work shows that natural genetic variation can explain lowly expressed genes that appear to be dosage compensated. Finally, based on our simulated data sets, we create guidelines for accurate differential expression analysis in trisomy cells, which we call trisomy-aware transcription analysis.

## Results

### A naive analysis suggests technical issues in dosage compensation detection

**Fig. 1:**
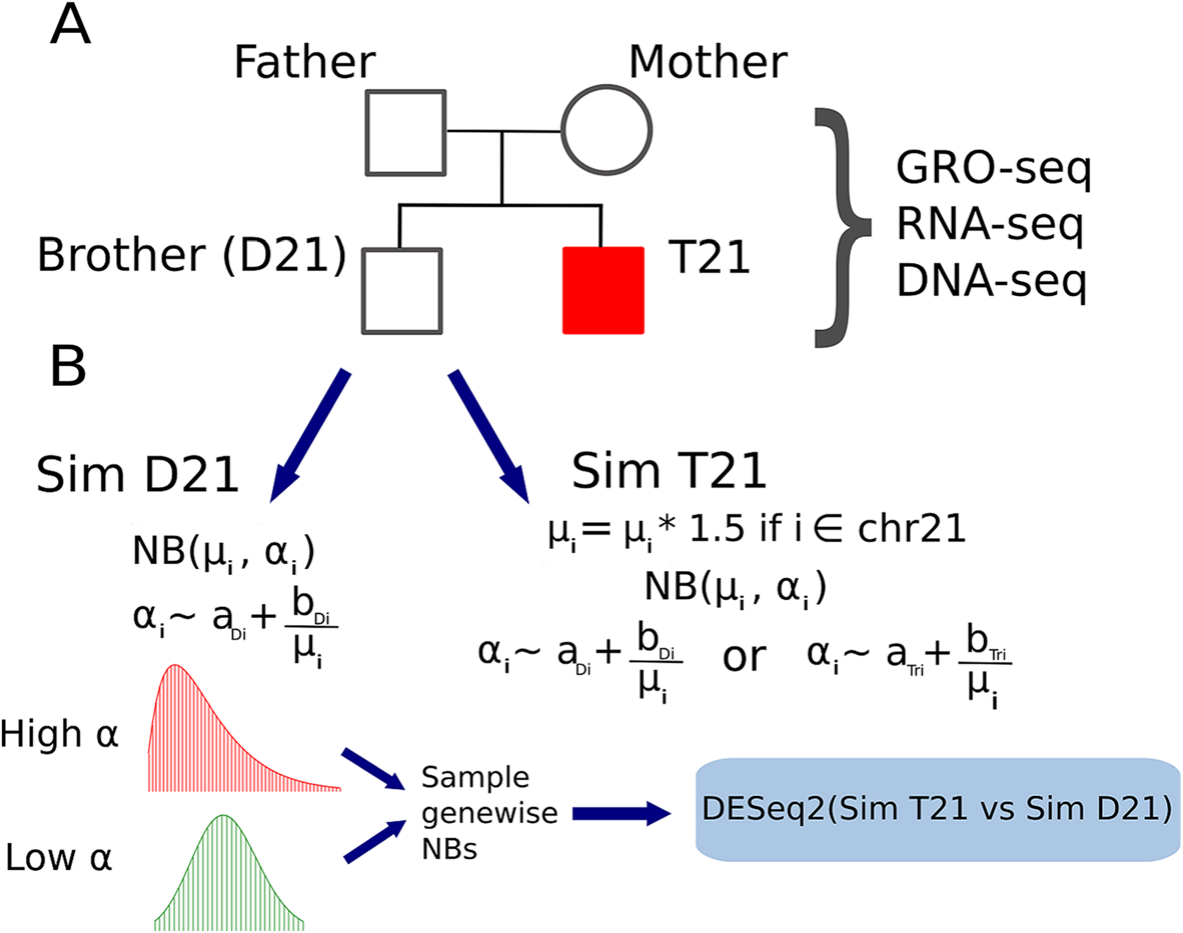
Summary of cell line generation and dataset simulation. **A** Pedigree depicting the relationship of our samples. Lymphoblastoid cell lines (LCLs) were derived from each of the individuals. Libraries for GRO-seq, RNA-seq, and DNA-seq were generated from these cell lines for downstream analysis. **B** Simulations generated from the D21 child. The RNA-seq datasets from this individual were averaged together to inform the mean counts (mu) for each gene i. The hyperparameters a (termed asymptotic dispersion) and b (termed extra-Poisson noise) are used to inform the gene-wise dispersion of each negative binomial (NB) distribution. New read datasets for each gene were then generated by random variate sampling from these distributions. For trisomic genes, the mean of the negative binomial distribution (represented as mu) is first multiplied by 1.5, ensuring that calculated fold change estimates between trisomic and disomic genes should yield an expected distribution around 1.5, modulated by dispersion. Varying hyperparameters were used to generate multiple simulated datasets

We sought to identify the sources of apparent dosage compensation, both technical and molecular. To this end, we focus on lymphoblastoid cell lines derived from a family of individuals where one child has Down syndrome (Fig. 1A). We reasoned that if dosage compensation did occur, it would either occur via inhibition of transcription or via an increase in the RNA degradation of that transcript. Thus, we examined both steady-state RNA levels via RNA-seq and nascent transcription with global run-on sequencing (GRO-seq), in triplicate (see the “Methods” section, Additional File 2: Supplemental Table 1 for sequencing information, Additional File 1: Figs. S1 and S2).

As an initial baseline, we first examined the typical differential analysis pipeline that leverages DESeq2 [22]. In this naive analysis, we make no adjustments to the defaults inherent to programs within the pipeline. Using the naive approach, we find the median fold change (MFC) of all genes on chromosome 21 in RNA-seq is 1.41, with 57.6% of these genes having a fold change below the expected 1.5-fold change. The trends in GRO-seq are similar, with an overall chromosome 21 median fold change of 1.38 and 48.8% of genes below the expected 1.5-fold change (Additional File 1: Fig. S3). Consistent with previous reports, we also observed many genes which were significantly above gene-dosage levels (e.g., interferon-related genes MX1 and MX2; see also Additional File 1: Figs. S4, S5, S6, S7).

To identify genes as dosage compensated, we must specify specific criteria — i.e., how much below the expected 1.5× levels is unusual? First, we considered a fold change cutoff. In this idealized case, if two populations of genes exist (i.e., dosage compensated and not compensated) one would expect to see two distinct but overlapping distributions of fold change in the data. To examine this, we generated a cumulative distribution function of fold change for all chromosome 21 genes in both RNA-seq and GRO-seq (Additional File 1: Fig. S3, red line). We noted that the majority of genes were distributed around 1.5; however, no apparent secondary population was immediately clear — instead, we observed a smooth distribution of fold changes below 1.5, suggesting fold change is distributed on a continuum. While this distribution does not in and of itself disprove the existence of transcription dosage compensation, these results argue that there is no obvious cutoff for identifying dosage-compensated genes. Indeed, these results are in agreement with Hwang et al. findings that dosage-compensated genes (defined in their approach as FDR *q*-value< 0.01) are rare in trisomy RNA-seq datasets [5]. This led to the question of how trisomy data influences differential expression and the assessment of dosage compensation. To address this question, we turn our attention to dissecting the typical analysis pipeline, using DESeq2 as a representative technique, in order to identify how T21 influenced these results.

### Simulations reveal the technical basis of reduced fold change calculations in trisomic datasets

To carefully assess the impact of trisomy data on the standard differential expression analysis, we need to know a priori the correct answer. To achieve this, we simulated T21 and D21 data sets, using the child with D21 data as a reference. Briefly, we used the D21 data along with parameters (such as variance) utilized by DESeq2 to create an artificial gene counts table (Fig. 1B, see the “Methods” section for full details). The simulated T21 individual was generated in the same way, but now all genes on chromosome 21 are at a 1.5× increase from the simulated D21 individual.

**Fig. 2:**
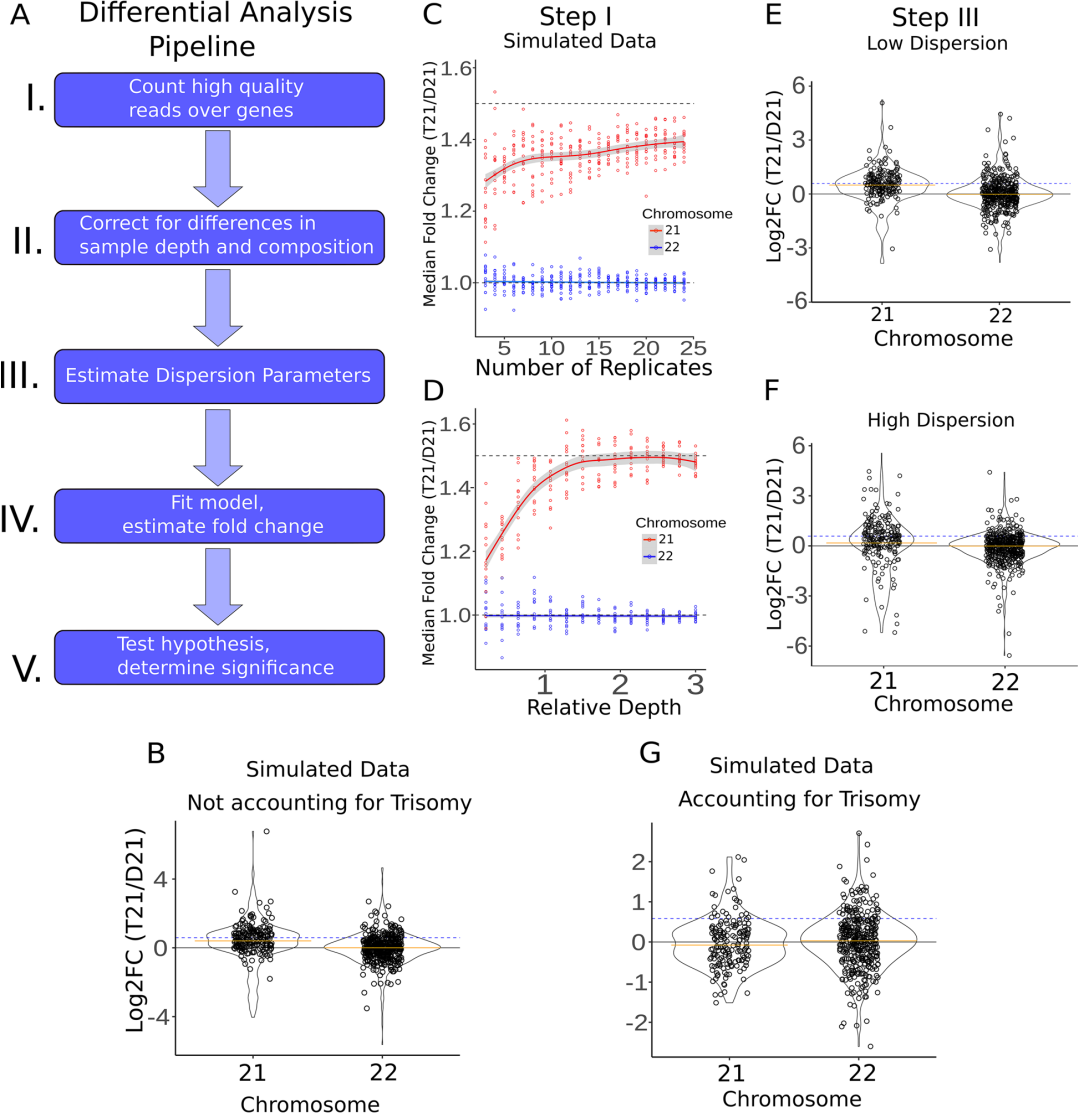
Fold change distributions of RNA-seq and GRO-seq datasets. **A** Pipeline of differential analysis. Variations at any step have the potential to increase or decrease fold change calculations for chromosome 21 genes (see also Additional File 1: Figs. S11, S15, S16). **B** Naive differential analysis of simulated T21 and D21 datasets using the similar dispersion parameters found in real data (asymptotic dispersion = 0.03, extra-Poisson noise = 3.5, see Additional File 1: Fig. S11). For chromosome 21 genes (which were simulated at 1.5×), the median fold change is 1.40. **C** Effects of shifting parameters of simulated datasets. Simulated datasets with varying numbers of replicates (asymptotic dispersion = .01, extra-Poisson noise = 1). **D** Simulated datasets with varying levels of depth (asymptotic dispersion = .01, extra-Poisson noise = 1). **E** Violin plots showing fold changes of simulated datasets when dispersion parameters are low (asymptotic dispersion = .01, extra-Poisson noise = 1). The orange line is the Median Fold Change (MFC). **F** Violin plots showing fold changes of simulated datasets when dispersion parameters are high (asymptotic dispersion = .05, extra-Poisson noise = 30). The orange line is the Mean Fold Change (MFC).** G** Simulated data violin plots showing fold changes after applying adjustments for each step in the pipeline. Results are consistent with no dosage compensation in T21 datasets in the simulated data

We then run the standard analysis pipeline (Fig. 2A) on the simulated data, calculating the fold change and its significance for all genes from each simulation. First, we run the simulation using the parameters from the naive analysis pipeline (Fig. 2B) and even though the chromosome 21 genes in the simulated data are at 1.5×, we observe distributions and median fold change similar to the biological data (MFC = 1.4). Next, we seek to isolate individual components of the differential expression pipeline (Fig. 2A) by running the simulation several times, modifying the count table’s read depth, replicate number, or variance to test each variable’s effect on both differential expression and fold change estimation. For simplicity, we present only the distributions for chromosome 21 (expected to be at 1.5×) and chromosome 22 (expected to be at 1×; all other disomic chromosomes showed the same results as chromosome 22).

#### Sequencing depth and read counting methodologies

Tools such as DESeq2 seek to quantify within-condition variability using principled models of read-count data to determine changes between conditions that exceed expected variability [16]. As such, the key first characteristics of sequencing data is the number of replicates available per condition. Therefore, we first examined the impact of replication, assuming initially that all replicates are of relatively high quality (low variance). To this end, we simulated data with varying replication, between 2 and 25 replicates per condition. We found that the median fold change estimation generally increased with additional replicates (Fig. 2C), consistent with the notion that additional replication is always a good strategy.

The parallel concern to replication is sequencing depth. It is important to note that nascent transcription typically has lower overall counts per gene than RNA-seq when the two protocols are sequenced to roughly equivalent depths. This arises because a much larger fraction of the genome is transcribed than is stable. Consistent with this, we noticed that low fold change estimates correlated with low expression levels (Additional File 1: Fig. S8) in actual data. Therefore, we next simulated datasets with varying depths, ranging from 0.1 times to 10 times the depth of the original data. We found that decreasing the depth of the simulated datasets resulted in a decreased fold change estimation for many genes, with concomitant reduced median fold change estimates (Fig. 2D). Thus, it is important to have adequate depth for accurate gene fold change estimates. However, additional sequencing is not an option when reanalyzing public data sets and in some cases, increased sequencing depth can be cost-prohibitive. Importantly, increased depth is not expected to fully alleviate the fold change estimation issue; as with increased depth, unexpressed genes are more likely to have reads assigned to them due to noise. Thus we suggest users employ a minimum coverage filter to remove low signal genes as potential false positives when determining which genes are potentially dosage compensated.

In these initial simulations, we noticed that genes with the most dramatic apparent dosage compensation in our naive analysis disappeared in the simulations. Specifically, highly expressed chromosome 21 genes with many genomic repeats, such as ribosomal genes, often appeared at typical expression levels in our real datasets (Additional File 1: Fig. S9), but not in our simulated data. This suggests that repeat regions shared between chromosome 21 and other chromosomes are sensitive to the mapping strategy. During mapping, these reads can be sponged away from chromosome 21 genes, resulting in a lower fold change estimation. This is, of course, dependent on the employed mapping strategy and how multiple mapped reads are handled. Combining a minimum read cutoff and masking repeat regions or removing multi-mapping reads effectively removed many of these genes as false positive dosage compensations. We suggest that users filter these genes from their list before dosage compensation analysis, either by masking repeat regions before counting reads or manually removing genes with a high number of genomic repeats before subsequent analysis [23].

#### Size factor calculation for sample normalization

After counting reads, the next step is to normalize the data between libraries (step II in Fig. 2A). Normalization accounts for differences in sequencing depth between samples and is crucial to proper differential analysis. DESeq2 utilizes a median-of-ratios method to find a normalizing “size factor” for each library [22]. In short, DESeq2 assumes that the majority of genes are similarly expressed from sample to sample. By calculating the ratio between the counts for a gene in one sample versus the mean count in all samples and then finding the median of these ratios, samples can be effectively normalized to the genes most likely to remain unchanged in all samples. We thus wondered if this normalization method was being influenced by the trisomic condition of one of our samples, where we expect a priori for a set of genes to be changed between the two samples simply because they reside on the aneuploid chromosome.

To investigate the impact of trisomy on size factor estimation, we removed chromosome 21 from both the actual and simulated data sets. In both cases, chromosome 21 genes had only a minimal effect on size factor calculation (Additional File 1: Fig. S10), consistent with the relatively small proportion of genes on chromosome 21 (approximately 1%) compared to the rest of the genome. Importantly, this result was robust to overall sequencing depth, which we showed by modulating the sequencing depth of our simulations. While the empirical result suggests including chromosome 21 genes in size factor estimation has little effect on the results, we nevertheless recommend removing chromosome 21 genes for the size factor calculation, as this is more consistent with the theory behind the median-of-ratios method.

#### Dispersion estimation and sample replication

We next investigated the effects of the model fitting process of DESeq2 on fold change estimation in T21 cells. In general, gene expression is estimated by fitting a negative binomial distribution with two parameters: the mean and the dispersion (both of which inform the variance of the distribution). Both values can be inferred directly by maximum likelihood estimation, which calculates the mean expression level for each group of replicates, and then determines the dispersion value. However, this method is susceptible to error at lowly expressed genes or when a low number of replicates are available, as systemic noise begins to dominate [22]. More optimal methods employ a Bayesian process allowing information sharing across multiple genes and replicates. In particular, DESeq2 assumes that genes with similar expression levels exhibit similar dispersion values. Information can thus be shared across genes and samples to more accurately estimate gene-wise dispersion, which increases the fidelity of fold change estimation and dispersion calls even within noisier data. However, if this assumption fails — i.e., if a cluster of similarly expressed genes has higher dispersion than expected – the resulting fits will not accurately reflect the underlying biology. We thus endeavored to answer this question: does the presence of a T21 sample affect the model estimation steps of differential analysis?

The default method used in DESeq2 for calculating gene-wise dispersion (step III in Fig. 2A) involves starting with the maximum likelihood estimate of dispersion, plotting these values against expression levels, and then fitting an asymptotic curve in the form of $y = a + b/x$ [22]. Here, *a* and *b* are the fitting parameters, *y* is the dispersion estimate, and *x* is the gene expression level. The parameters *a* and *b* control the two ends of the curve; for low-expression genes, the parameter *b* will be more meaningful, leading to higher dispersion estimates for the fitted curve at these genes, a phenomenon we refer to as extra-Poisson noise. For high-expression genes, the *b*/*x* term trends toward 0, and thus the fitted curve will asymptotically approach the value of parameter *a*, a trend we refer to as asymptotic dispersion. The resulting fitted curve is then used to inform one more round of dispersion estimation, effectively shrinking gene-wise dispersion towards the fitted curve. An increase in either parameter increases the value of the initial dispersion estimate. Still, the effects are felt asymmetrically depending on the expression level and the amount of information available to each gene (i.e., replicates and sequencing depth).

To determine the effects of T21 on dispersion estimation, we extracted the fitting parameters used in dispersion estimation from our biological data sets, with and without chromosome 21 genes. As a control, we also compared removing an equivalent number of random genes from the other chromosomes (see the “Methods” section). We observed an increase in both fitting parameters relating to gene-wise dispersion when trisomic genes were included (Additional File 1: Fig. S11), leading to the question: how does this increase in the initial dispersion fit affect differential calls and fold change estimates?

To address this question, we generated data sets sampled from a negative binomial distribution with the same means but varying dispersion values for chromosome 21 genes in the T21 simulations. When we compared across a wide range of dispersion estimates, we noted higher dispersion parameters resulted in decreased fold change estimations for chromosome 21 genes (Fig. 2E, F; Additional File 1: Fig. S12). Specifically, the distribution of fold change drastically shifted towards 1.0, albeit with a broader spread (MFC = 1.28). The effects of higher dispersion on fold change estimation could be partially offset in simulated data by adding more replicates and depth (Additional File 1: Fig. S13), consistent with the fact that more replication and read depth are critical to overcoming the effects of systemic noise. Notably, the fact that a high number of replicates creates a MFC closer to 1.5× may also contribute to the disparate results regarding dosage compensation reported in previous studies; large-scale studies that integrate several available data sets result in more confident fold change estimation indicating no dosage compensation (i.e., most genes are at the expected 1.5× fold change) [5].

#### Fold change shrinkage and hypothesis testing

The final steps of differential expression analysis are fold change estimation and hypothesis testing. Here, DESeq2 provides the option to utilize a Bayesian method for fold change estimation, using a prior distribution centered around a fold change of 1.0, which effectively shrinks fold change estimates towards 1.0. As with dispersion estimation, the resulting shrinkage effect is more substantial for low-expression genes. These estimates (known as maximum a posteriori or MAP estimates) are generally considered more reliable than MLE calculations of fold change for low expression genes [22]. However, this assumption can fail if the prior distribution does not represent the underlying biology, as is true for trisomic genes (Additional File 1: Fig. S15). Researchers who utilize MAP estimates will thus note more genes that appear dosage compensated, although this apparent “compensation” is mainly due to the shrinkage effects of the prior distribution. In general, we recommend users exercise caution when interpreting MAP estimates as evidence for dosage compensation; genes that experience strong fold-change shrinkage should be filtered out from analysis, or MLE calculations should be used instead.

Hypothesis testing in the standard differential analysis pipeline uses the default null hypothesis that each gene’s expression levels are equal in both groups. Given that chromosome 21 genes exist in three copies and DESeq2 seeks to identify deviations from typical, one might expect most of chromosome 21 to be differentially expressed, e.g., statistically significant. However, when we ran our simulation using parameters similar to the real data (Fig. 2B, *p*adj $< .01$), no genes on chromosome 21 are called as statistically significant. This arises because while the median fold change of chromosome 21 encoded genes is elevated (MFC = 1.4), the dispersion genome wide is high enough that this change is not deemed significant. In our simulations, we altered dispersion over a broad range (Additional File 1: Fig. S12) and while the median fold change varied (Fig. 2E, F), no genes on chromosome 21 were deemed statistically significant. In contrast, increasing replicates and read depth, even with higher dispersion values, did yield 126/262 differentially expressed chromosome 21 genes in RNA-seq simulations (MFC = 1.43) (Additional File 1: Figs. S13, S14). In all cases, significant genes showed fold change estimates near or greater than the expected 1.5-fold. Thus, with high replication DESeq2 detects the typical 1.5× transcription levels of chromosome 21 as statistically significant, but finds no genes with lower-than-expected expression levels.

Arguably, this arises because differential expression analysis is a distinct question from identifying dosage compensation. The default null hypothesis used in hypothesis testing is incorrect for identifying dosage-compensated genes, as all of chromosome 21 is expected to be elevated. Consequently, to identify dosage compensation using the standard differential expression pipeline it is necessary to either adjust the null hypothesis or the input data.

The first method is to change the fold-change threshold for the hypothesis tests of chromosome 21 genes from the default value of 1 to the dosage-informed value of 1.5, such that significant gene calls deviate from the DNA dosage-informed expectation. Under these tests, significant gene calls below a fold change of 1.5 are potential candidates for dosage compensation. In both our actual and simulated data, most chromosome 21 genes are not considered below levels expected by gene dosage when using this method (Additional File 1: Fig. S16). However, we note that this method cannot reliably utilize the MAP estimates of fold change, as the prior distribution does not reflect the new alternative hypothesis.

The second method, which we prefer, is to perform an additional normalization step before the differential analysis. In DESeq2, the ploidy number of each gene in each sample can be loaded into a normalizing matrix. The resulting read counts are normalized both by the library’s size factor and the gene’s ploidy number. Subsequent fold change shrinkage and hypothesis testing can thus utilize the default parameters, as even trisomic genes are expected to exhibit a fold change of 1.0 under these conditions (Fig. 2G, Additional File 1: Fig. S17). Thus, significant genes with a fold change of less than 1.0 are candidate dosage-compensated genes. Given we simulated data with a 1.5-fold change, this approach correctly finds no significant chromosome 21 genes in our simulated data, regardless of the dispersion parameters used (Additional File 1: Fig. S18).

**Fig. 3:**
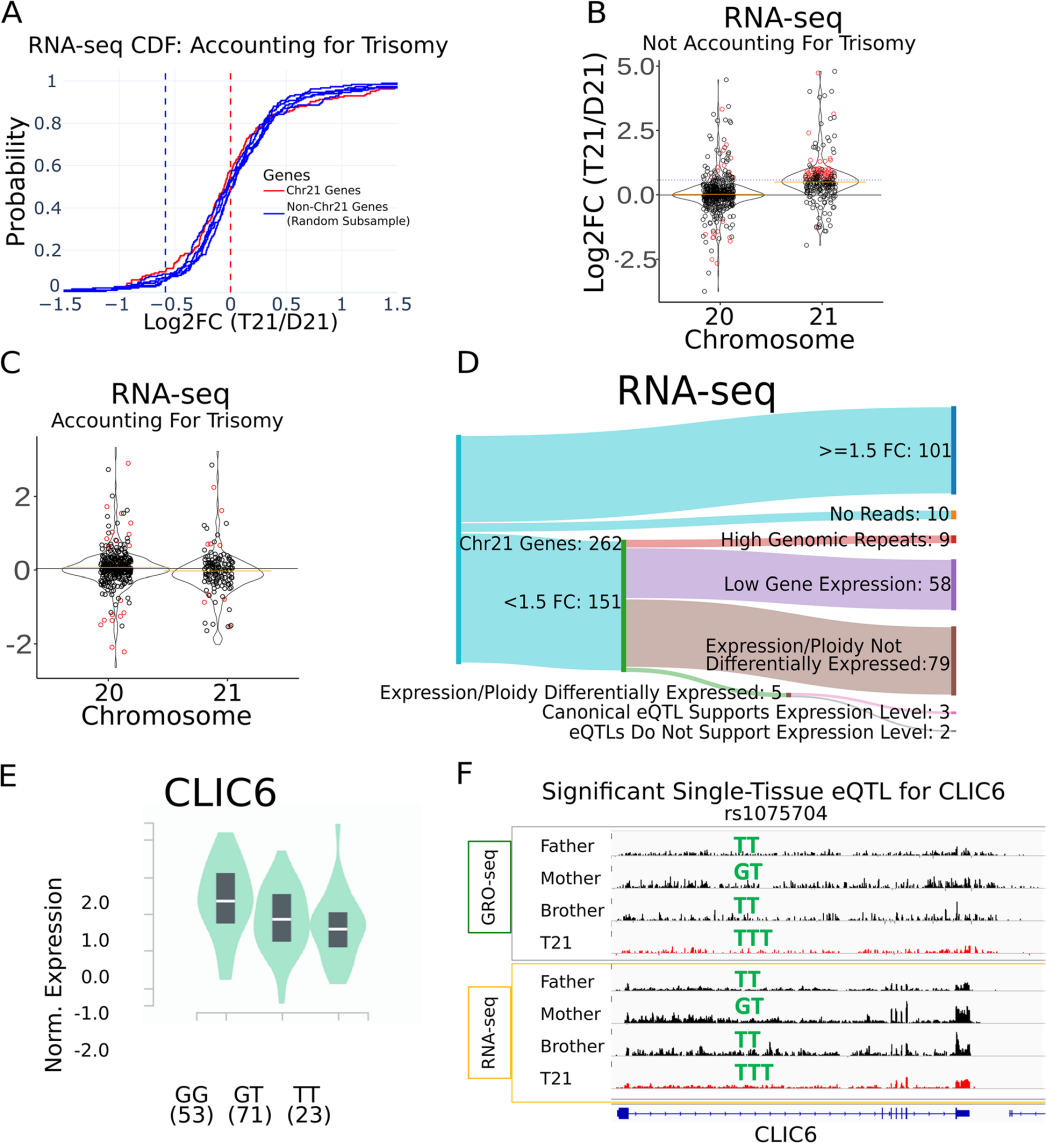
Alternative explanations to disparate fold change estimates. **A** Cumulative distribution plot of fold changes found in real RNA-seq data, after accounting for trisomy. The solid red line indicates all chromosome 21 genes. Each solid blue line is a randomly selected set of genes from all other chromosomes. **B** Violin plots indicating Log2 fold change between T21 and D21 samples. Significant gene calls are colored red (*p*adj $< .01$). **C** Same as **B**, but using a trisomy-aware pipeline for analysis. **D** Sankey diagram depicted the filtering process of our RNA-seq analysis. The initial 151/262 genes identified as potentially dosage compensated can alternatively be explained by genomic repeats, high variance from low expression genes, or technical artifacts related to failing to normalize the data to the ploidy number. The remaining genes can be explained by the presence of eQTLs (see also Additional File 1: Fig. S22)). **E** Example boxplot indicating relative expression of the gene CLIC6 with one eQTL. **F** Genome viewer tracks for the gene CLIC6 for all four family members, in GRO-seq (top) and RNA-seq (bottom). The T21 track is indicated in red. The allelic makeup of the eQTL in **E** is indicated by the green text above each track

In biological data, ploidy normalization brings the distribution of chromosome 21 fold change estimates in line with other chromosomes (Fig. 3A–C, Additional File 1: Fig. S19). Thus chromosome 21 genes exhibited an MFC of 0.96 with dosage normalization in RNA-seq (Fig. 3C). Furthermore, 1009 genes were considered differentially expressed between the two brothers, of which only five were on chromosome 21 (out of 143 total chromosomes 21 genes, after read count filtering). In GRO-seq analysis, chromosome 21 genes had an MFC of 0.97 and 3820 genes were differentially expressed, of which 20 genes fell on chromosome 21 (out of 144 total chromosome 21 genes, after filtering) (Additional File 1: Fig. S20). We note that the proportion of significant genes on chromosome 21 is similar to those obtained when we compare two disomic individuals; the distribution of fold changes and the number of differential genes are consistent so long as the reads are normalized by the ploidy number (Additional File 1: Fig. S21). Normalizing by ploidy is additionally advantageous, as MAP estimates of fold change can be utilized for visualization and downstream analysis. Furthermore, subsequent power analyses are more relevant to trisomic data once the counts have been adjusted [24].

In all, our simulations led us to the construction of a differential analysis pipeline more suited to trisomic samples. First, we find that multi-mapped reads can cause a handful of genes to appear dosage-compensated; we thus suggest removing these reads prior to analysis. Next, we note that low expression genes are especially prone to appearing dosage compensated; we thus suggest employing a minimum coverage filter to avoid these false positives. Finally, our trisomic samples appeared to be noisier relative to their disomic counterparts; this variance resulted in lower fold change estimations for many chromosome 21 genes. As such, we suggest either employing a normalization matrix to adjust all gene counts by their ploidy number or using the appropriate null hypothesis to determine potential dosage compensation in trisomy. Rather than relying on cutoffs, our trisomy-aware pipeline uses DESeq2’s significance calls to determine which gene expression levels are significantly different than their expected values based on gene dosage (Fig. 3C, D, Additional File 1: Fig. S22). Furthermore, by accounting for the genes which contribute the most variance in fold-change calculations, we remove likely false positives from the analysis. We thus contend that our pipeline is a more rigorous determinant for dosage-compensated genes which properly accounts for the underlying variance of the data.

### Reduced fold change on chromosome 21 are consistent with identified eQTLs

After adjusting the data analysis pipeline to be trisomy-aware, we conclude that nearly all chromosome 21 encoded gene transcription levels (GRO-seq) and expression levels (RNA-seq) are proportional to DNA dosage(Additional File 1: Fig. S18). However, a small number of genes on chromosome 21 remain potentially dosage compensated, as precisely five genes in RNA-seq and 20 genes in nascent transcription (Fig. 3D, Additional File 1: Fig. S20) have lower-than-expected levels.

Given the small number of genes remaining and the lack of known dosage compensation mechanisms in humans, we reasoned that there might be a genetic basis for the reduced expression of these genes. More concretely, we hypothesize that apparent dosage compensation could arise from allele-specific sequence variation. To explore this possibility, we sought to compare the genome sequence of these individuals to known expression quantitative trait loci (eQTL) where a specific sequence variant is associated with lower expression within the population [25]. Thus we performed whole genome DNA sequencing on the cell lines of all four family members (see the “Methods” section for full details; Additional File 2: Supplemental Table 1 for sequencing information).

We used the GATK package to call single-nucleotide polymorphisms (SNPs) in each member of the quartet [21]. Briefly, reads were mapped and realigned before variants were called using Haplotype caller (see the “Methods” section for full description). Because chromosome 21 is triploid in the individual with Down syndrome, Haplotype caller was used twice — once with the default ploidy of two and once with ploidy set to three. Variants called using the ploidy of three versions were kept only for chromosome 21 in the individual with Down syndrome; otherwise, the default calls were used.

We reasoned that any genetic variation that reduces chromosome 21 gene expression in the general population could be present in our T21 sample, leading to reduced expression levels in the individual. To test this hypothesis, we compared genome variations identified in each individual to previously identified eQTLs [21, 26]. We limited our search to eQTLs identified in either lymphoblastoid cell lines or their nearest related tissue (whole blood). For this analysis, we required the variant to be identifiable in at least one of the parent samples as well as the child with trisomy 21. In RNA-seq, we found that the reduced expression of three of the five apparently dosage-compensated genes (CLIC6, ITSN1, C2CD2) could potentially be explained by known expression controlling polymorphisms (Fig. 3D, Additional File 3: Supplemental Table 2). For example, the rs1075704 eQTL exists in the human population as a GG, GT, or TT (Fig. 3E), and the T allele correlates with lower expression of CLIC6 in lymphoblastoid cells. This SNP, rs1075704, shows variable CLIC6 gene expression across the disomic samples in both our RNA-seq and GRO-seq (Fig. 3F). In our trisomic sample, CLIC6 has an expression level less than 1.5× the average of all disomics, with the genotype TTT. So the lower-than-expected expression observed at CLIC6 can be explained by the genotype, which is not considered by the typical differential expression pipeline. Consistent with the allele identity, these three genes also have lower-than-expected transcription in GRO-seq.

We also reasoned that the eQTL data in GTEx may be incomplete, that there may exist other alleles that lead to reductions in expression data. To explore this possibility, we next compared the two parents to each other, reasoning that differences observed between the parents could be subsequently inherited by either of the two children. Using this technique, we found that 9 of the remaining 17 genes in GRO-seq were also differentially transcribed between the parental samples, suggesting the lower than expected levels in the trisomy sample may be modulated by an inherited parental haplotype (Additional File 1: Fig. S22). Altogether, we found reasonable explanations for most genes (60%) which fell below expected levels in T21 (Fig. 3). These results were consistent in both RNA- and GRO-seq (Additional File 1: Fig. S22).

## Discussion

We sought to add clarity to the conflicting reports in the literature concerning whether molecular dosage compensation occurs within individuals with Down syndrome [5, 10–15]. Our results uniformly suggest that dosage compensation in T21 is rare, if not completely absent, in transcriptomics data, both nascent and steady-state RNA. Using simulated data, we found that computational pipelines developed for disomic samples could lead to erroneous conclusions on trisomy data, and this likely contributes to confusion in the literature. We were able to use our simulated data to create a trisomy-adjusted differential expression analysis pipeline that correctly estimates the simulated fold change of chromosome 21 genes (see the “Methods” section for a summary of pipeline adjustments). When we applied this modified pipeline to our actual samples, most of the apparent dosage compensation was lost. Importantly, the remaining genes with lower-than-expected expression were predominantly attributable to alleles with reduced expression levels. Thus our work agrees with other recent studies suggesting no reduction in RNA expression levels via dosage compensation in T21 [5] and provides explanations regarding previous reports to the contrary. In addition, we have extended the conclusion of no dosage compensation to nascent RNA transcription.

While we focused here on dosage compensation, our findings have implications for using trisomic samples in differential expression analysis. We have shown that the inclusion of trisomic samples in a differential expression analysis pipeline can inflate the dispersion of the data if not adequately accounted for. The presence of trisomic samples in a data collection can affect parameter estimation within the differential analysis pipeline, even when the trisomic sample is not part of the final comparison. For example, comparing the son with D21 to his father results in two sets of significant gene calls, depending on whether the trisomic sample is included in the upstream processes (Additional File 1: Fig. S21).

We also note that, by design our study did not compare large groups of individuals with and without T21. In any study, genes below expected expression levels could arise from trisomy 21-altered pathways, molecular dosage compensation or genetic allele frequency differences. We used a family of related individuals to minimize allele variation and focus specifically on molecular dosage compensation. We found no molecular dosage compensation but we did find allele variation that could be driving lower-than-expected expression levels. In a larger cohort of unrelated individuals with Down syndrome, some alleles may have lower-than-expected expression levels. Consistent with this, a recent study of large groups of unrelated disomic and trisomic individuals found many genes are expressed below a 1.5-fold change expectation [27].

As there are many genes with sequence variations that lead to higher or lower expression [21, 26], our work leads to speculation about whether highly expressed alleles could be selected against in a Down syndrome background. More than 75% of trisomy 21 embryos are lost due to spontaneous abortion and it is currently unclear why [27]. If genes on chromosome 21 exist that are deleterious pre-birth when expressed at high levels, individuals with T21 harboring three copies of these alleles would be more likely to be lost. This would result in allele bias within the population of live individuals with T21, favoring the lower expressed allele. It stands to reason that this selection would lead to an over-representation of low expression alleles in the Down syndrome population. Considering the allele-specific expression levels of genes will be a useful avenue for understanding any unique selection pressures existing within a Down syndrome population.

Assessing whether particular alleles have a skewed frequency in the T21 population relative to typical individuals requires large numbers of genomes from both typicals and individuals with Down syndrome. As an alternative, an extensive collection of T21 RNA-seq could be leveraged toward identifying allele bias. In recent years, the allelic fold change method has been implemented for quantifying eQTL effects [28]. While this model is currently constrained to disomic samples only (i.e., it only allows for three different allelic combinations), the model could be extended (allow for a fourth allelic state) and applied to trisomic samples. As such, population eQTL data could be used to identify lower-than-expected fold change at some alleles in Down syndrome. It remains to be seen whether any chromosome 21 allele bias exists in individuals with T21 [12].

Finally, we note our work was limited to only the exploration of dosage compensation at the transcription level and does not account for potential changes at the protein level due to aneuploidy. Indeed, recent studies identify several protein complexes which are potentially dosage compensated in Down syndrome, likely due to a change in stoichiometric ratios of their respective subunits [29]. Furthermore, new research contends that a majority of proteins undergo dosage compensation in other aneuploidies, such as those found in common cancers [30]. In any case, mRNA abundance is not fully predictive of protein abundance, and conclusions regarding transcription dosage compensation cannot necessarily be extended to the protein level, or absolute quantification methods used to assess protein abundance.

## Methods

### Cell culture of LCLs

The lymphoblastoid cell lines of 4 individuals were obtained from the Translational Nexus Biobank (COMIRB 08-1276), University of Colorado School of Medicine, JFK Partners. The Translational Nexus Biobank acts as the honest broker for the samples used for this study and samples were provided to the study team in a deidentified manner. As such, this study is considered non-human subjects research, and additional IRB approval was not required. Lymphoblastoid cell lines (LCLs) were seeded in upright T-25 suspension flasks with 10 ml RPMI (10% FBS, 1X L-glutamine, 1X penicillin/streptomycin). These were passaged approximately every 2 to 3 days by pelleting the cells via centrifugation (300 × g, 5 min) and resuspension. Cells were grown to an approximate density of 1 million cells per ml, before being harvested for subsequent experiments. The three RNA-seq replicates per sample were based on three separate growths of cells and the three GRO-seq replicates per sample were based on three separate growths. The correlation between replicates can be seen in Additional File 1: Fig. S2.

### Nuclei isolation

Cell cultures were collected in 50 ml Falcon tubes and centrifuged in a fixed-angle rotor centrifuge at 300 × g, 4 °C, for 5 min. The supernatant was poured off, and 10 ml ice-cold PBS was added to resuspend the cell pellet. The previous spin and PBS wash were repeated 2 more times. Cells were then resuspended in 10 ml ice-cold Lysis Buffer (10 mM Tris-HCl pH 7.5, 2 mM MgCl$_2$, 3 mM CaCl$_2$, 0.5% IGEPAL, 10% glycerol, 2 U/mL SUPERase-IN, brought to 10 ml with 0.1% DEPC DI-water). The resuspended cells were incubated for 10 min on ice. Nuclei were then centrifuged in a fixed-angle rotor centrifuge at 1000 × g for 10 min at 4 °C. The resulting supernatant was poured off, and the pellet was resuspended with 1 ml lysis buffer, using a wide-mouth P1000 pipette tip. The volume of was brought to 10 ml with lysis buffer, and centrifuged for 1000 × g, 4 °C, 5 min, in a fixed-angle rotor centrifuge. The lysis buffer wash was repeated as above. Nuclei were then resuspended in 1 ml freezing buffer (50 mM Tris-HCl pH 8.3, 5 mM MgCl$_2$, 40% glycerol, 0.1 mM EDTA pH 8.0, brought to volume with 0.1% DEPC DI-water), and transferred to a 1.7-ml Eppendorf tube. Nuclei were pelleted at 1000 × g, 4 °C, 5 min. The resulting supernatant was removed by pipetting, and the pellet resuspended with 500 μl freezing buffer. The nuclei were again pelleted at 1000 × g, 4 °C, 5 min. The supernatant was removed, and the nuclei were resuspended in 110 μl freezing buffer and stored at − 80$^\circ$C until library preparation.

### GRO-seq and library preparation methods

Wash solutions for anti-BrdU-beads were prepared ahead of time: binding buffer (0.25X SSPE, 37.5 mM NaCl, 0.05% Tween, 1mM EDTA pH 8, 0.2% SuperRNAseIN), low salt wash buffer (0.25X SSPE, 0.05% Tween, 1mM EDTA pH 8, 0.2% SuperRNAseIN), high salt wash buffer (0.25X SSPE, 137.5mM NaCl, 0.05% Tween, 1 mM EDTA pH 8, 0.2% SuperRNAseIN), TET buffer (10 mM Tris-Cl pH 8.0, 0.05% Tween, 1 mM EDTA pH 8.0, 0.2% SuperRNAseIN), elution buffer (150 mM NaCl, 50 mM Tris-Cl pH 7.5, 20 mM DTT, 0.1% SDS, 1 mM EDTA, 0.2% SuperRNAseIN).

Anti-BrdU beads (Santa Cruz, sc-32323-ac) were prepared by washing and blocking. Per sample, 60 μl were washed twice in 500 μl binding buffer. beads were blocked in 500 μl blocking buffer (1X binding buffer, 0.1% PVP, 1 μg/mL BSA UltraPure, 0.002 more superRNAseIN). Beads were then resuspended in 450 μl binding buffer.

Run-on reactions were performed as in [31]. In brief, ice-cold isolated nuclei (100 μL) were added to 37 °C 100 μL reaction buffer (final concentration: 5 mM Tris-Cl pH 8.0, 2.5 mM MgCl$_2$, 0.5 mM DTT, 150 mM KCl, 10 units of SUPERase In, 0.5% sarkosyl, 500 μM rATP, rGTP, and Bromo-UTP, 2 μM rCTP). The reaction was allowed to proceed for 10 min at 37 °C, followed by the addition of 500 μL of TRIzol LS. RNA was extracted once with phenol-chloroform, washed once with chloroform, and precipitated with 3 volumes of ice-cold ethanol and 1–2 μL GlycoBlue. The pellet was washed in 75% ethanol before resuspending in 18 μL of DEPC-treated water.

Libraries were prepared similar to [32]. In brief, RNA was treated with 2 μl NEB fragmentation buffer at 94° for 5 min. The RNA was then buffer exchanged via BioRad P-30 (or a G-25) column per the manufacturer’s protocol. Next, 2 μl DNaseI and 5 μl of 10X RQ1 DNase buffer and water were added to create a 1X final concentration of the buffer. After incubation at 37 °C for 10min, 5 μl DNAse stop solution was added, and the reaction was placed at 65 °C for 5 min. As prepared above, the beads in 450 μl binding buffer were immediately added to this mixture.

Fragmented nascent RNA was purified using Anti-BrdU beads via washing with 500 μl for 1 min with each solution (binding buffer, low salt buffer, high salt buffer, TET buffer). Between washes, beads were spun down per manufacturer instructions. RNA was eluted from the bead via soaking the beads in 125 μl elution buffer at 42 °C, 10 min 2 times. The beads were then added to acid phenol-chloroform, as was the elute. The RNA was washed with chloroform and precipitated with 5M NaCl and 3× the ethanol column. The second round of anti-BrdU bead binding and extraction enriched BrdU-labeled products was completed as above. NEBNext Ultra II RNA was used to transform the nascent RNA into an RNA-seq library. The product was amplified 15 ± three cycles and products $>150$ bp (insert $> 70$ bp) were size selected with 1X AMPure XP beads (Beckman) before being sequenced.

### RNA-seq

RNA was isolated from the cells via TRIzol extraction. NEBNext rRNA Depletion kit was used to remove rRNA. NEBNext Ultra II RNA was used to transform the RNA into an RNA-seq library. The product was amplified 15 ± three cycles and products $>150$ bp (insert $> 70$ bp) were size selected with 1X AMPure XP beads (Beckman) before being sequenced.

### Mapping and visualization of RNA datasets

The fastq files for GRO-seq and RNA-seq were trimmed and mapped to the GRCh38/hg38 reference genome and prepared for analysis and visualization through our in-house pipelines. In short, resulting fastq read files were first trimmed using bbduk (version 38.05) to remove adapter sequences, as well as short or low quality reads. Reads were mapped with HISAT2 (version 2.1.0), and resulting SAM files converted to BAM files with Samtools (version 1.8). Multimapped reads were filtered from these files. BedGraph files were generated using Bedtools (version 2.25.0), and converted to TDF files for visualization in IGV using IGVtools (version 2.3.75). Quality metrics were generated with FastQC (version 0.11.8), Preseq (version 2.0.3), RSeQC (version 3.0.0). Figures were generated through MultiQC (version 1.6).

### Differential expression analysis

Differential transcription was performed using the DESeq2 (version 1.26.0) R package (R version 3.6.3). Gene counts were generated using featureCounts (version 1.6.2) from the R Subread package (version 1.6.0), counting over the gene body region (+150 from transcription start site to annotated transcription end site) to avoid the 5′ peak. In both RNA-seq and GRO-seq analyses, reads were counted over the gene (including intronic regions), to ensure that results between these two protocols should be comparable. Annotations were downloaded from RefSeq (release number 109, downloaded August 14, 2019, from UCSC genome browser). Only annotations with both RNA-seq and GRO-seq signals were considered, again to keep both analyses comparable. For featureCounts, BED6 region files were converted to SAF format with the following command: awk -F “\t” -v OFS=“\t” ‘print{$4, $1, $2, $3, $6}’ region.bed > region.saf. Only the highest transcribed isoform of each gene was considered.

For our corrected analysis, we made use of DESeq2’s normMatrix parameter. The normalization matrix was generated by assigning each gene in the analysis its ploidy number divided by 2. For all genes not on chromosome 21, this number was thus 1. For genes on chromosome 21 samples in trisomy, this number was 1.5. We also removed reads within regions of genomic repeats, set the betaPrior parameter to false, and set an expression level cutoff at the second quintile of the baseMean counts for all genes. River plots were generated using the web interface of the program Sankeymatic (https://github.com/nowthis/sankeymatic.git).

The script and full table of results are available at the github repository listed below.

### Simulation of trisomy and disomy datasets

Reads were simulated using the negative binomial model as part of the scipy (version 1.8.0) Python package (version 3.6.3). The negative binomial means for each gene were estimated by averaging the counts across replicates for the D21 child samples in RNA-seq. For the disomic simulations, these means were used to directly inform the negative binomial parameters. For the trisomic simulations, the means for chromosome 21 genes were first multiplied by 1.5, to simulate the expected increase in dosage. The negative binomial instance is parameterized as follows:

1 $$\begin{aligned} NB(n,p): p=\mu /\sigma ^2 \end{aligned}$$

2 $$\begin{aligned} n=\mu ^2/\sigma ^2-\mu \end{aligned}$$

3 $$\begin{aligned} \sigma ^2=\mu +\alpha *\mu ^2 \end{aligned}$$

4 $$\begin{aligned} \alpha \sim a + b/\mu \end{aligned}$$

where *a* and *b* are controllable hyperparameters. For the disomic genes, we used values of .01 and 1 for *a* and *b*, respectively. For trisomic genes, we varied these values from .001 to 1.2 (for *a*) and 1 to 100 (for *b*). The negative binomials were also scaled based on the depth of the original biological samples, from 0.1 times the depth to 3 times the depth. Each simulated counts file was generated with a minimum of three replicates for both the disomy 21 and trisomy 21 samples. For details, see the github repository for these scripts.

### Whole genome sequencing

DNA was prepared using a variety of methods then sequenced on a Highseq 2000 to an approximate depth of 40× (see table fastq for full details). The father, mother, and individual with Down syndrome were first sequenced at Illumina and received as one bam file per person, then split by read group (RG) into individual lane bam files using samtools (version 0.1.19) view. Those files were then converted to fastq using bedtools (version 2.16.2) bamtofastq. All other sample files were received as fastq files. Each fastq file was mapped to hg38 using bowtie(version 2.0.2) with the setting — very-sensitive and using options to retain both library preparation and sequencing reaction information. Tracking sample-level information is essential for detecting and removing individual sequencing reaction error rates. SAM files were converted to sorted BAM files using samtools (version 1.2) view and sort. All files for one individual were merged using Picard tools (version 1.72) MergeSamFiles. BAM files were sorted and duplicates were marked using Picard tools (version 1.72) SortSam and MarkDuplicates.

### Whole genome variant calling

GATK (version 3.3-0) was used for variant calling. BAM files were realigned using IndelRealigner with optional flags -known Mills_and_1000G_gold_standard.indels.hg38.vcf -known 1000G_phase1.indels.hg38.vcf [Note: the realignment table was created using all the merged files with RealignerTargetCreator optional flags -known Mills_and_1000G_gold_standard.indels.hg38.vcf -known 1000G_phase1.indels.hg38.vcf]. Then, BaseRecalibrator and PrintReads was used to recalibrate the bases (optional arguments -knownSites Mills_and_1000G_gold_standard.indels.hg38.vcf -knownSites 1000G_phase1.indels.hg38.vcf -knownSites dbsnp_138.hg38.vcf). Haplotypes where called two times, via HaplotypeCaller; once with the optional flags -nct 4 –emitRefConfidence GVCF –dbsnp dbsnp_138.hg38.vcf –variant_index_type LINEAR –variant_index_parameter 128000 and another time with those flags and the flag -ploidy 3. Then vcf files were created for each trio (mother, father, child) using GenotypeGVCFs. To create a vcf that contained chr21 as triploid in only the individual with Down syndrome we created our own code in Python to combine the two types of family vcf files. This program combined the family vcf files by taking any lines that started with “chr21_” or “chr21” from the vcf files with the triploid child variants, and all lines that did not start with “chr21_” or “chr21” from the family diploid vcf files. The vcf file is provided at the Zenodo link listed below.

### GTEx datasets/SNP analysis

We downloaded the GTEx (version 8) database of eQTLs and their associated genes for all available tissues [26]. We merged these databases and filtered out all SNPs identified in our DNA-seq experiments that were not present in the merged GTEx database. We compared the expected effects of each SNP from GTEx with the allelic ratios of each of our datasets. We then asked whether there was at least one SNP that could explain the expression/transcription levels we observed in the real data when each parent was compared to the child with T21. The scripts and full results table are available at the github repository listed below.

### Summary of differential analysis pipeline adjustments

Many analysis pipelines (including the popular DESeq2 algorithm) use a null hypothesis that the fold change between two samples is 1, and are thus the default analysis is ill-equipped for fold change estimation and differential analysis when DNA dosage suggests an alternative fold change is expected. These native settings can be easily adjusted, leading to a more reliable analysis. In summary:

1. Apply a minimum read coverage filter, depending on read depth (30 used in this study)
2. Mask repeat regions or remove multi-mapping reads for read counting
3. Remove chromosome 21 genes for size factor calculation
4. For noisy samples, increase sequencing depth or replication
5. Adjust the null hypothesis or normalize read count by ploidy number

### Supplementary information

**Additional file 1:** **Figure S1.** Sequencing Dataset QC Metrics. **Figure S2.** Sample Distance Heatmaps. **Figure S3.** CDF Plots of Fold-Changes. **Figure S4.** Heatmap of Gene Transcription in GRO-seq. **Figure S5.** Heatmap of Gene Expression in RNA-seq. **Figure S6.** Volcano Plots of Gene Transcription in GRO-seq. **Figure S7.** Volcano Plots of Gene Expression in RNA-seq. **Figure S8.** Violin Plots of Expression by Percentile. **Figure S9.** Effects of Repeat Regions and Multi-Mapped Reads. **Figure S10.** Simulations of Size Factor Calculation. **Figure S11.** Dispersion Parameters of GRO-seq and RNA-seq. **Figure S12.** Effects of Dispersion on Fold-change Estimation. **Figure S13.** Effects of Replication and Depth. **Figure S14.** Power Analysis of Dosage Compensation. **Figure S15.** MLE and MAP Estimates of Fold-change. **Figure S16.** Utilizing Other Alternative Hypotheses. **Figure S17.** Violin Plots of Expression by Percentile After Adjusting Analysis Pipeline. **Figure S18.** Effects of Dispersion on Fold-change Estimation After Adjusting Analysis Pipeline. **Figure S19.** CDF Plots of Fold-change After Adjusting Analysis Pipeline. **Figure S20.** GRO-seq Violin Plots After Adjusting Analysis Pipeline. **Figure S21.** UpSet Plots for Significance Calls. **Figure S22.** GRO-seq Sankey Diagram.

**Additional file 2:** **Supplemental Table 1.**  DNA-seq Sample Information.

**Additional file 3:** **Supplemental Table 2.**  Identified SNPs and Corresponding Gene/eQTL Pairs.

## Data Availability

DNA sequence is available at the SRA with accession number PRJNA966937 [33], RNA-seq and GRO-seq data sets are available within GEO with accession number GSE230999 [34]. Genome output and input tables, including vcf are available at doi: 10.5281/zenodo.7527453 [35]. RNA-seq dataset mapping information is available at doi: 10.5281/zenodo.7527541 [36]. GRO-seq dataset mapping information is available at doi: 10.5281/zenodo.7527537 [37]. The GTEx version 8 database of eQTLs titled GTEx Analysis V8 was downloaded from the url: https://gtexportal.org/home/datasets on August 26, 2019 [21]. dbSNP database build version 138 was downloaded from ftp://gsapubftp-anonymous@ftp.broadinstitute.org/bundle/hg38/ on January 14, 2021 [25]. Analysis output tables, including output of differential expression analysis (naive and corrected) and GTEx comparison table are available in the github along with all scripts and analysis pipelines utilized. The github repository can be found at https://github.com/Dowell-Lab/DS_Normalization under the tag BMCbio2023 [38].

## Abbreviations

T21: Trisomy 21
D21: Disomy 21
DS: Down syndrome
GRO-seq: Global run-on sequencing
LCL: Lymphoblastoid cell line
SNP: Single nucleotide polymorphism
eQTL: Expression quantitative trait locus
MFC: Median fold change
MLE: Maximum likelihood estimation
MAP: Maximum a posteriori
