# Supplementary material for: Transcription dosage compensation does not occur in Down syndrome
Source: BMC Biol. 2023 Nov 10;21:228. doi: 10.1186/s12915-023-01700-4 (PMC10636926; doi:10.1186/s12915-023-01700-4)
Supplement: Supplementary file 1 — Additional file 1: Figure S1. Sequencing Dataset QC Metrics. Figure S2. Sample Distance Heatmaps. Figure S3. CDF Plots of Fold-Changes. Figure S4. Heatmap of Gene Transcription in GRO-seq. Figure S5. Heatmap of Gene Expression in RNA-seq. Figure S6. Volcano Plots of Gene Transcription in GRO-seq. Figure S7. Volcano Plots of Gene Expression in RNA-seq. Figure S8. Violin Plots of Expression by Percentile. Figure S9. Effects of Repeat Regions and Multi-Mapped Reads. Figure S10. Simulations of Size Factor Calculation. Figure S11. Dispersion Parameters of GRO-seq and RNA-seq. Figure S12. Effects of Dispersion on Fold-change Estimation. Figure S13. Effects of Replication and Depth. Figure S14. Power Analysis of Dosage Compensation. Figure S15. MLE and MAP Estimates of Fold-change. Figure S16. Utilizing Other Alternative Hypotheses. Figure S17. Violin Plots of Expression by Percentile After Adjusting Analysis Pipeline. Figure S18. Effects of Dispersion on Fold-change Estimation After Adjusting Analysis Pipeline. Figure S19. CDF Plots of Fold-change After Adjusting Analysis Pipeline. Figure S20. GRO-seq Violin Plots After Adjusting Analysis Pipeline. Figure S21. UpSet Plots for Significance Calls. Figure S22. GRO-seq Sankey Diagram. [file 12915_2023_1700_MOESM1_ESM.pdf]

Additional File 1: Supplemental Figures S1-S22

Differential Expression Analysis in Trisomy 21

Samuel Hunter, Jo Hendrix, Justin Freeman, Robin D. Dowell, Mary A. Allen*

∗ Corresponding author: mary.a.allen@colorado.edu

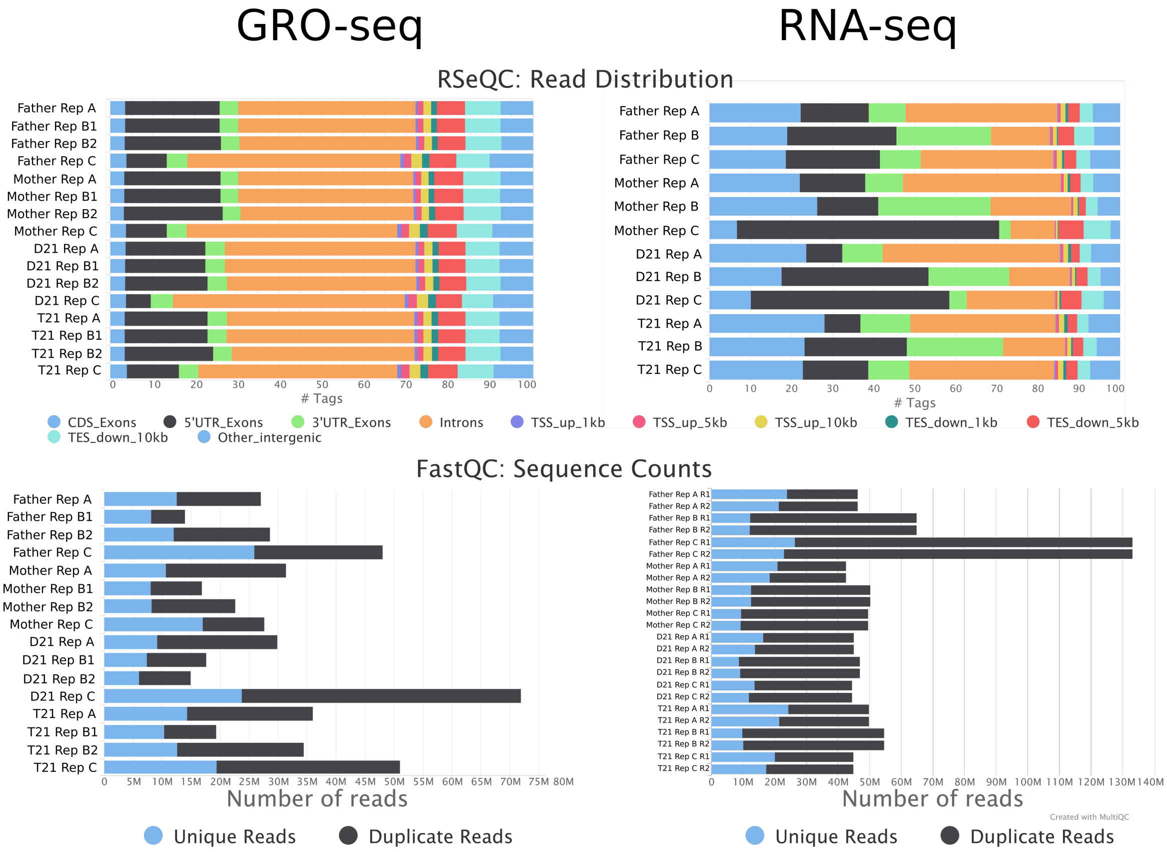

Fig S 1: **QC metrics of RNA-seq and GRO-seq datasets.** (Top) RSeQC read distribution plots of all datasets, showing relative abundance of reads at each listed genomic feature. (Bottom) FastQC plots showing number of total reads, and proportion of duplicate reads for each dataset.

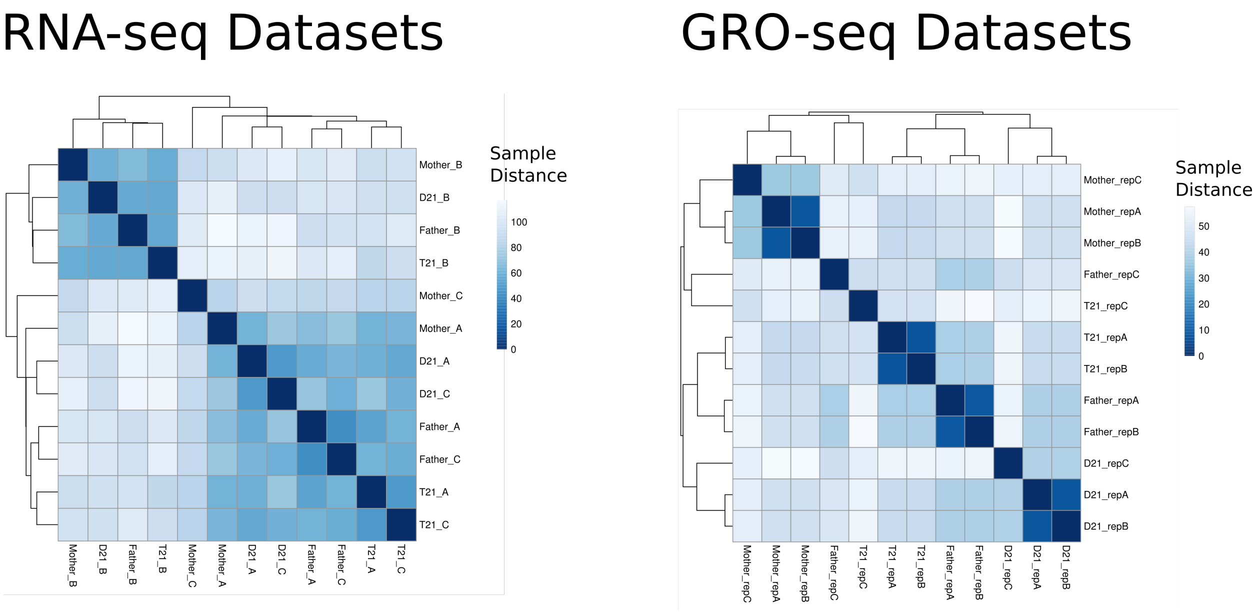

Fig S 2: **Sample Distance between GRO-seq and RNA-seq datasets** Euclidean distance between (Left) RNA-seq datasets (Right) GRO-seq datasets, including replicates. The letters A, B, C refer to biological replicates. For more information on samples, see the methods section.

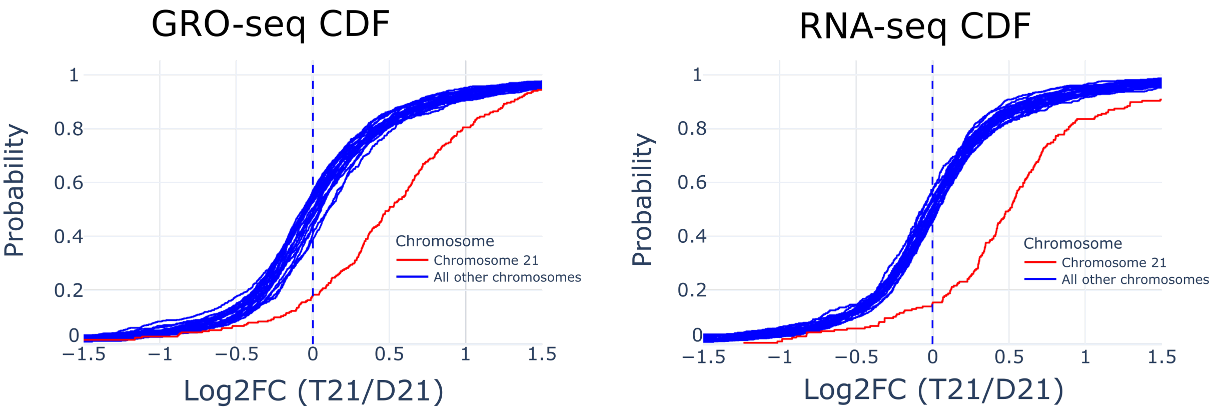

Fig S 3: **Cumulative distribution plot of fold changes of each chromosome.** All diploid chromosomes in blue; chromosome 21 in red. Dosage compensated genes are often identified using a fold change cutoff, indicated by each vertical dotted line (red:1.5x fold-change, blue: 1,0x fold-change); however, the reduced fold change estimations of these genes can also be explained by biological or technical variance. Left: GRO-seq. Right: RNA-seq.

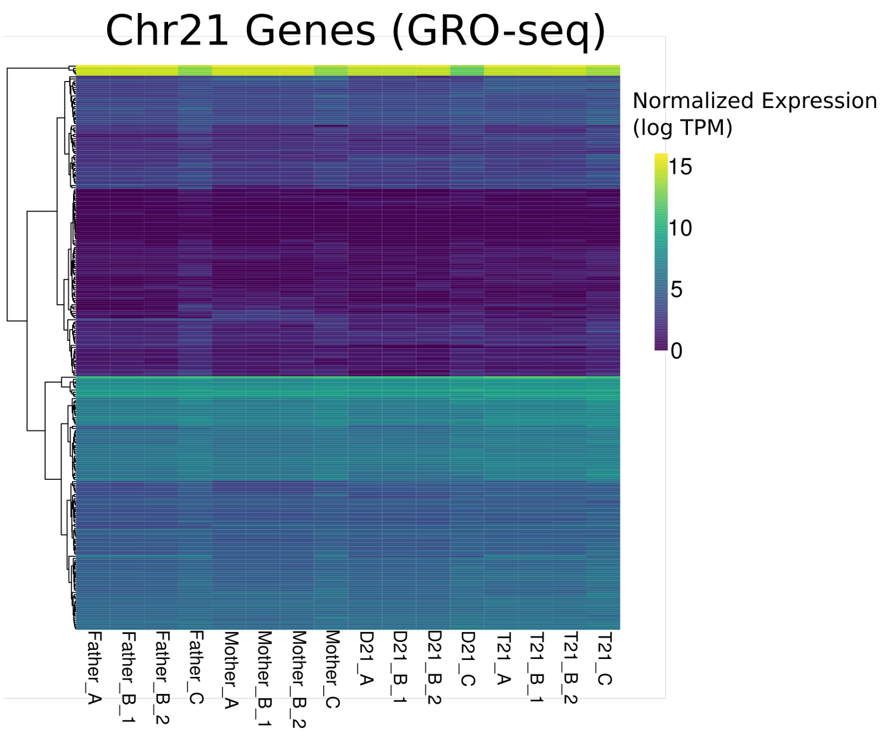

Fig S 4: **Heatmap of transcription levels of chromosome 21 genes in GRO-seq**. Counts are from raw mapped reads (includes multi-mapped reads), as log(TPM) value.

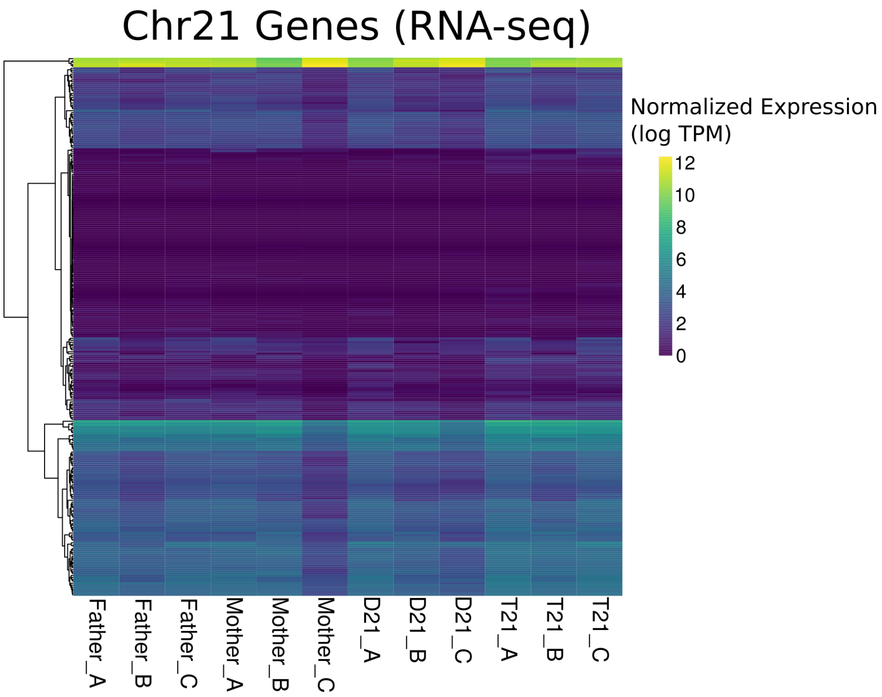

Fig S 5: **Heatmap of expression levels of chromosome 21 genes in RNA-seq**. Counts are from raw mapped reads (includes multi-mapped reads), as log(TPM) value.

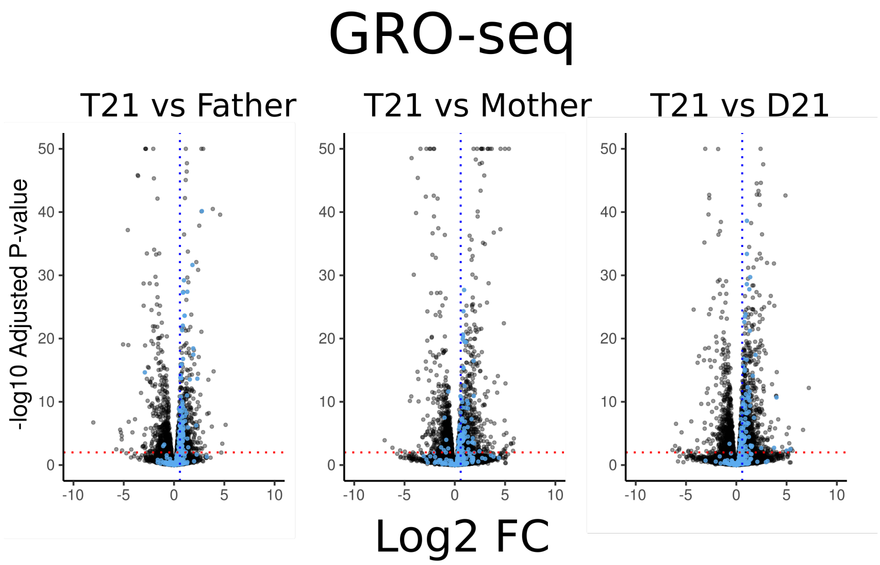

Fig S 6: **Volcano Plots of GRO-seq results**. Chromosome 21 genes are indicated in blue. LFC = 0.58 indicated by the blue vertical dotted line. Padj = 0.01 indicated by the red horizontal dotted line. Counts are from raw mapped reads (includes multi-mapped reads).

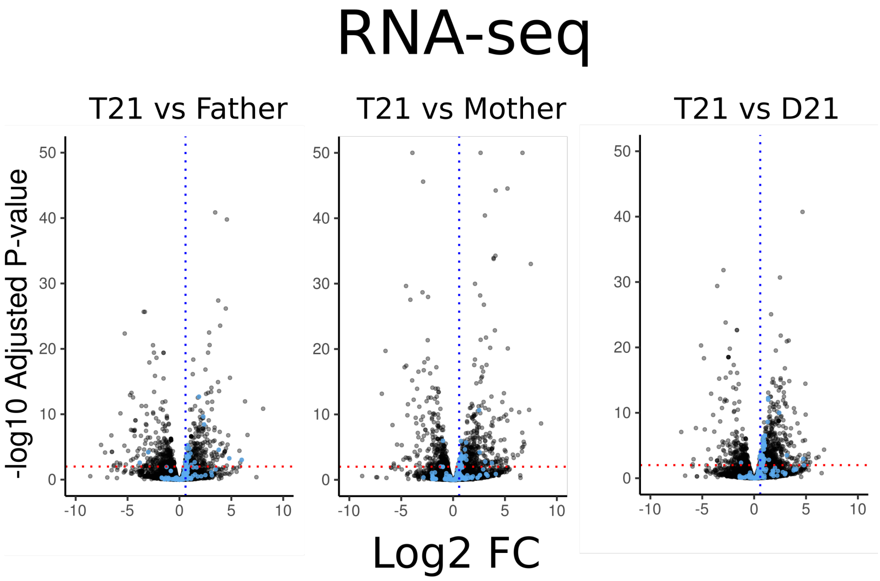

Fig S 7: **Volcano Plots of RNA-seq results**. Chromosome 21 genes are indicated in blue. LFC = 0.58 indicated by the blue vertical dotted line. Padj = 0.01 indicated by the red horizontal dotted line. Counts are from raw mapped reads (includes multi-mapped reads).

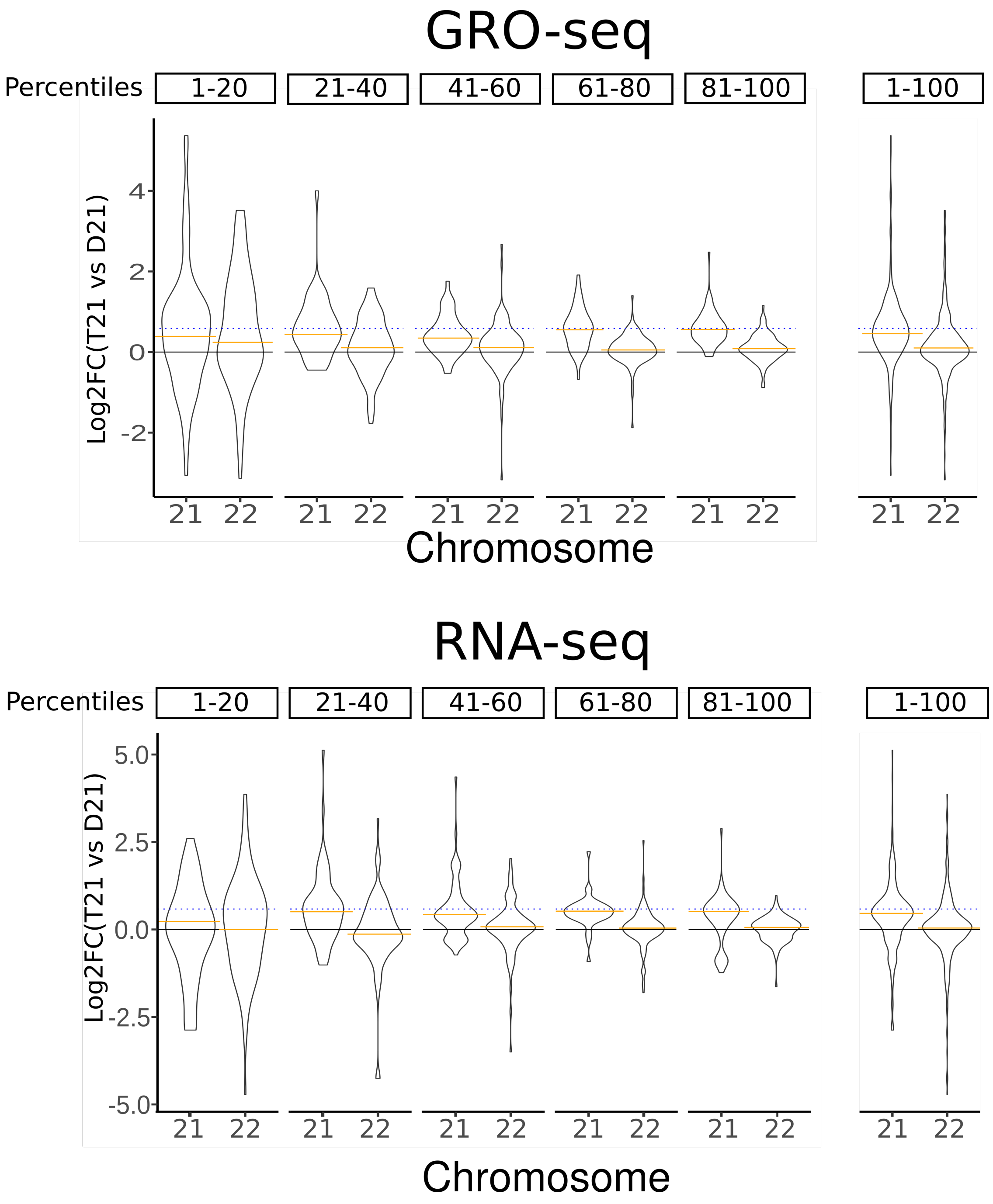

Fig S 8: **Fold change estimations (T21 vs D21) across expression levels in GRO-seq (top) and RNA-seq (bottom).** Genes are grouped by expression quantiles. Lower quantiles, on the left, contain the genes with the lowest expression (and therefore the genes with the highest technical variability in measurements). Median fold changes for each group indicated with an orange line. The lowest expression quantile has a noticeably lower fold change estimate for chromosome 21 genes. On the far right (labeled 1-100), the figure shows all genes in one violin plot regardless of expression levels.

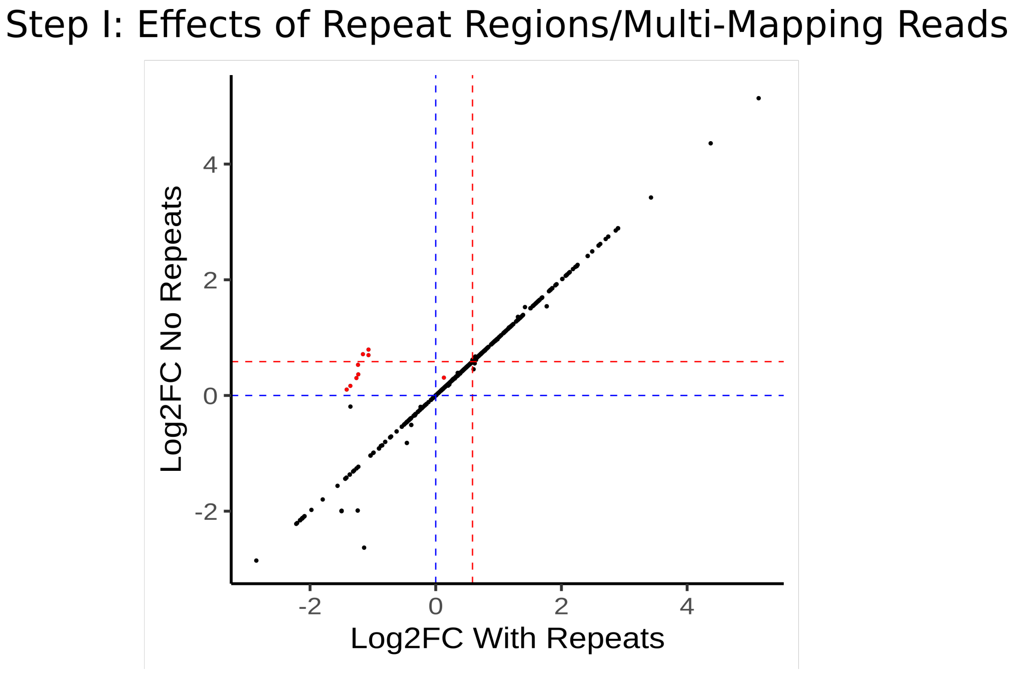

Fig S 9: **Effects of genomic repeats and multi-mapping reads on fold change estimates (T21 vs D21, RNA-seq).** Read counts were generated for all genes on chromosome 21, from bam files including genomic repeats and multi-mapping reads (x-axis), and bam files excluding these reads (y-axis). Nine genes show a reduced fold change estimation when repeat reads are included (highlighted in red). Red dashed lines indicate 1.5x fold change. Blue dashed lines indicate 1.0 fold change. The rRNA genes are highly transcribed from several locations within the genome (rDNA copies are located on chr13, chr14, chr15, chr21, chr22). Therefore, the fold change calculated when including multi-mapped reads is likely incorrect.

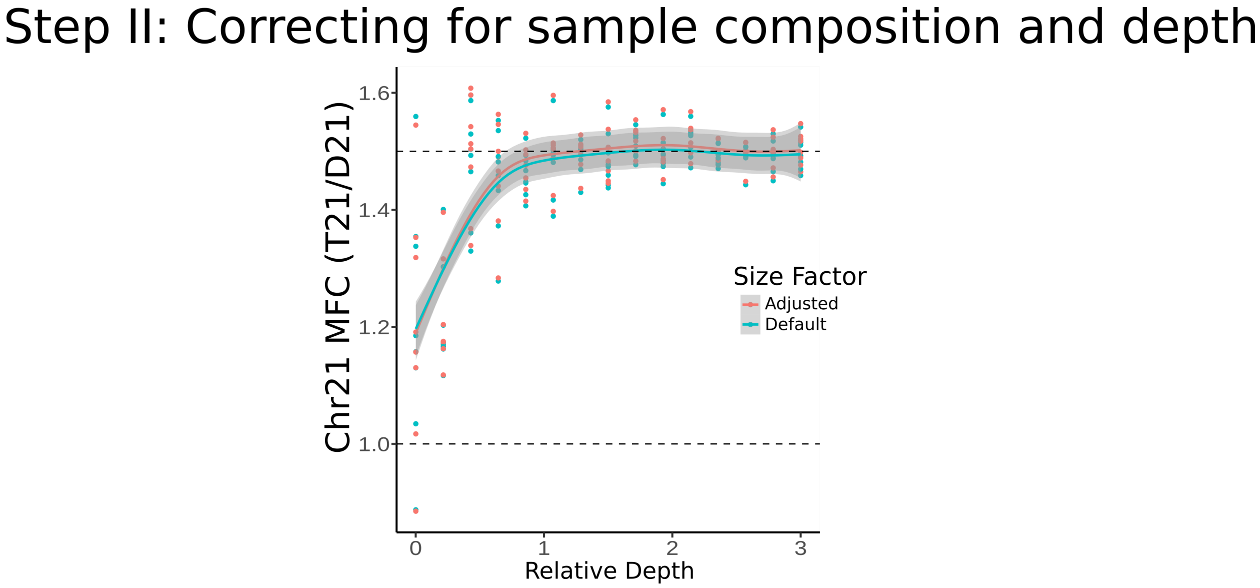

Fig S 10: **Fold change estimations in simulated data sets using two different size factor calculations.** Default (teal line): chromosome 21 genes are included in size factor calculation. Adjusted (red line): chromosome 21 genes are excluded from size factor calculation. In general, the differences in fold change estimates between these two approaches is minimal.

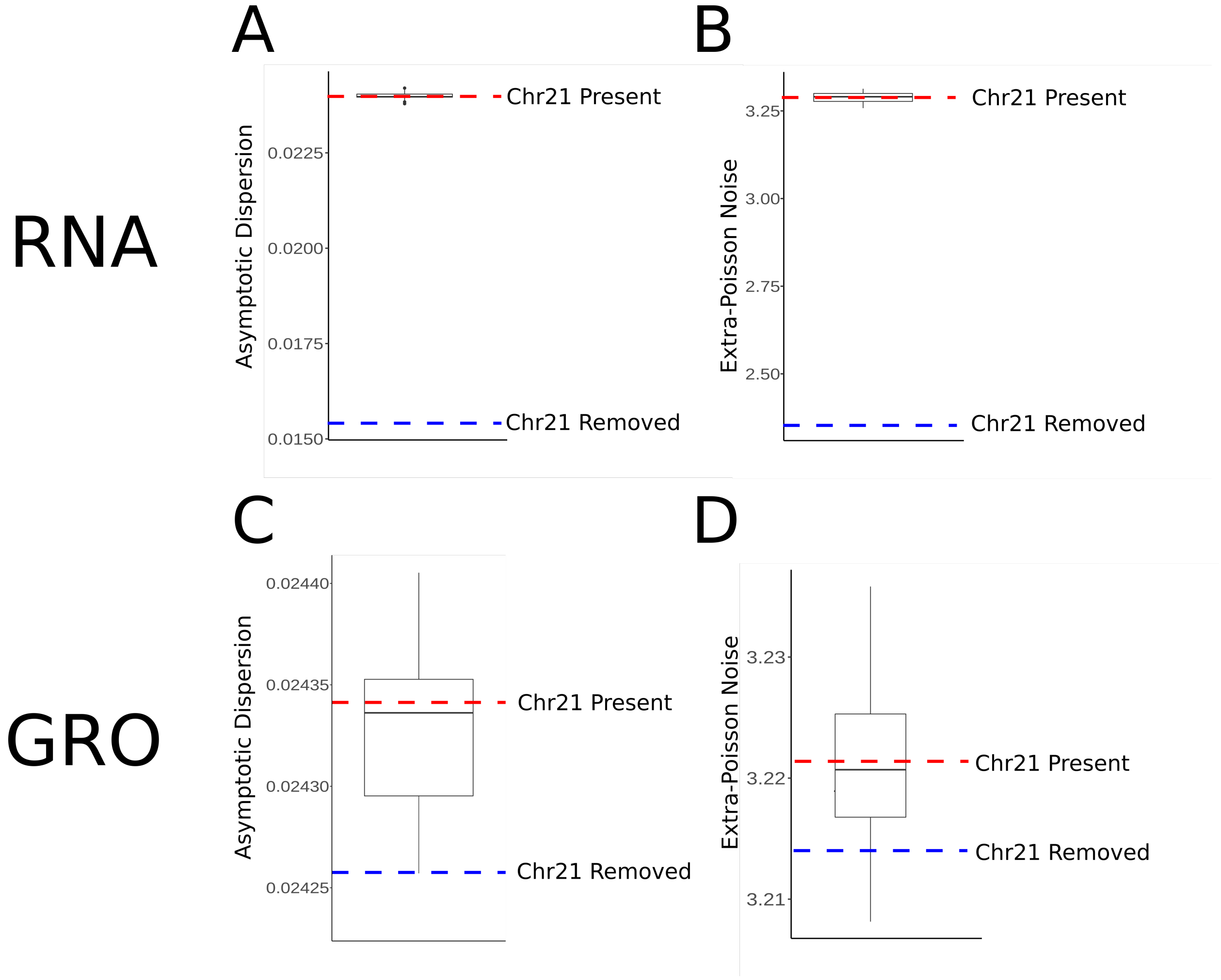

Fig S 11: **Dispersion fitting estimates calculated by DESeq2 with and without chromosome 21 genes.** Estimates are calculated including chromosome 21 genes (red) and excluding chromosome 21 genes (blue) for both RNA-seq (top) and GRO-seq (bottom). Specifically: (A) Asymptotic dispersion estimates in RNA-seq data, (B) extra-Poisson noise estimates in RNA-seq, (C) asymptotic dispersion estimates in GRO-seq data, and (D) extra-Poisson noise estimates in GRO-seq data. Additionally, an equivalent number of random non-chromosome 21 genes were excluded as a control (boxplots), showing that both values are slightly higher when chr21 is included. See Materials and Methods for information about how these values are estimated.

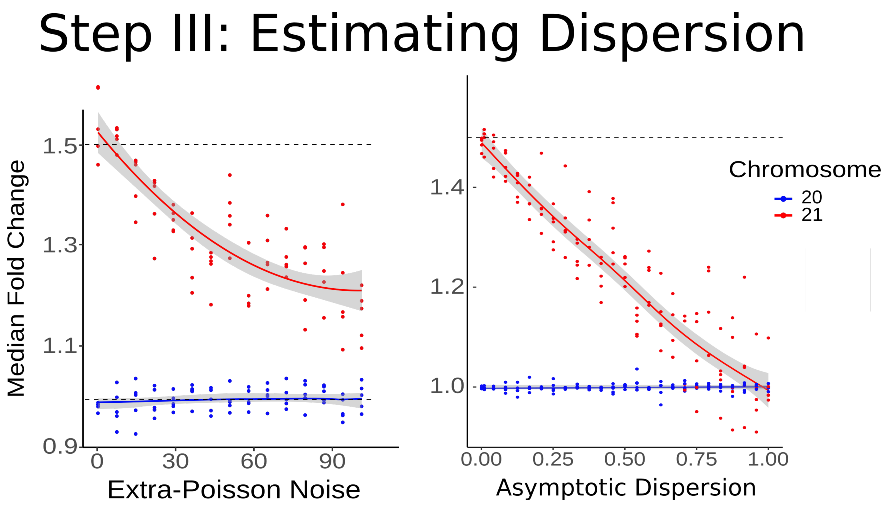

Fig S 12: **Scatterplots indicating the effects of increasing dispersion parameters on median fold change calculation in simulated data.** (Left) Effects of increasing extra-Poisson noise (asymptotic dispersion = 0.01). (Right) Effects of increasing asymptotic dispersion (extra-Poisson noise = 1). See Materials and Methods for details on simulating data.

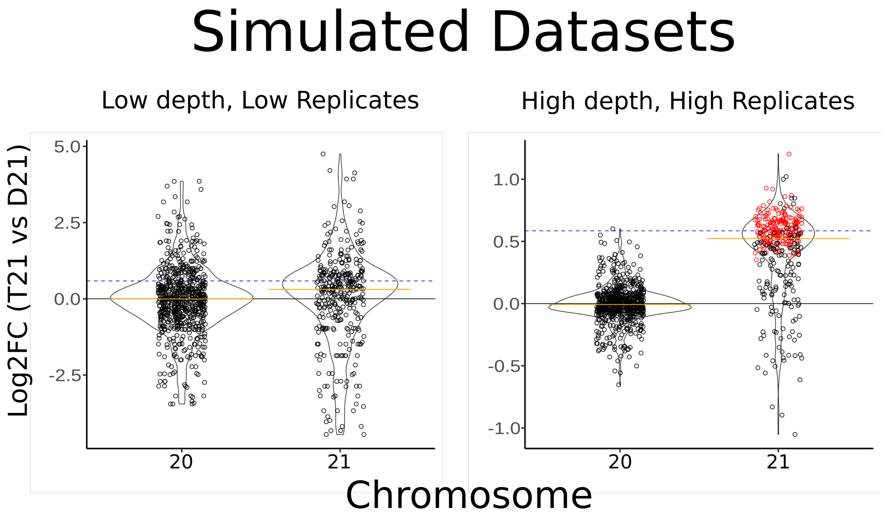

Fig S 13: **Fold-change distributions of simulated T21 and D21 data sets with high dispersion, varying the depth and replication number of the samples.** Simulations used high dispersion estimates (asymptotic dispersion=.08, extra-Poisson noise=8) and depth was changed relative to our D21 RNA-seq data. Median fold changes indicated with orange lines. (see also Supplemental Fig 1). Left: low depth (1x) and low replication (n=3). Right: high depth (3x) and high replication (n=12).

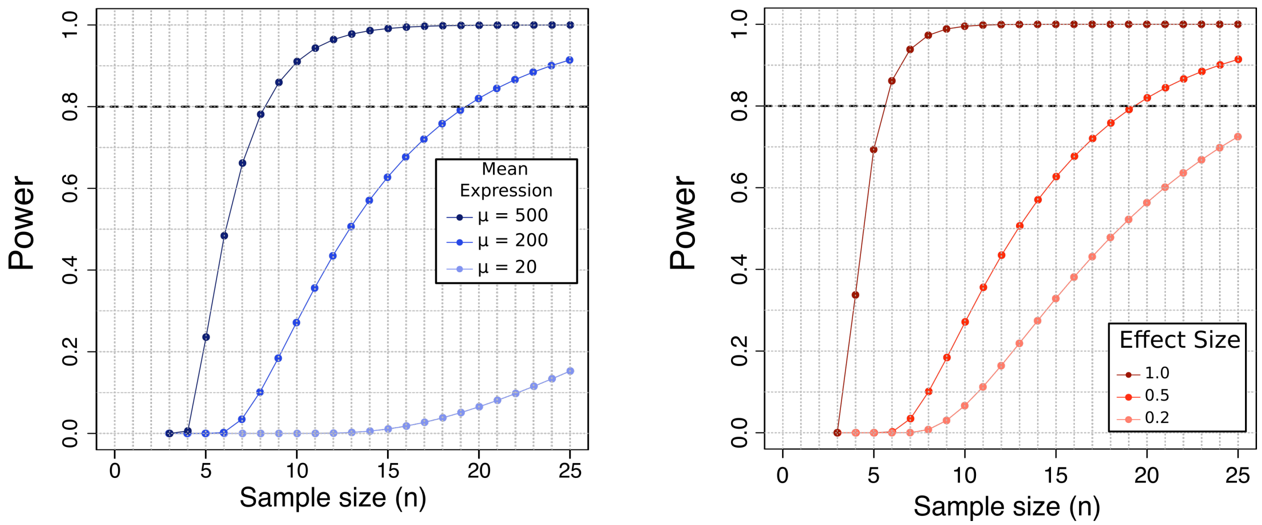

Fig S 14: **Power analysis of a "dosage-compensated" gene using dispersion parameters of normalized trisomic data**. The proportion of unchanging (non-compensated) genes was set at 0.9. Dispersion was estimated as in DESeq2 (a + b /μ, estimated here at a = 0.03 and b = 5). (Left) Power analysis with different mean expression levels, denoted by μ, with an effect size of 0.5 (a fold change of 1.5, or a Log2 fold-change of +/- 0.58). (Right) Power analysis with differing effect sizes, with a mean expression level μ= 200. Power graphs generated from the R library ssizeRNA (v1.3.2) [1].

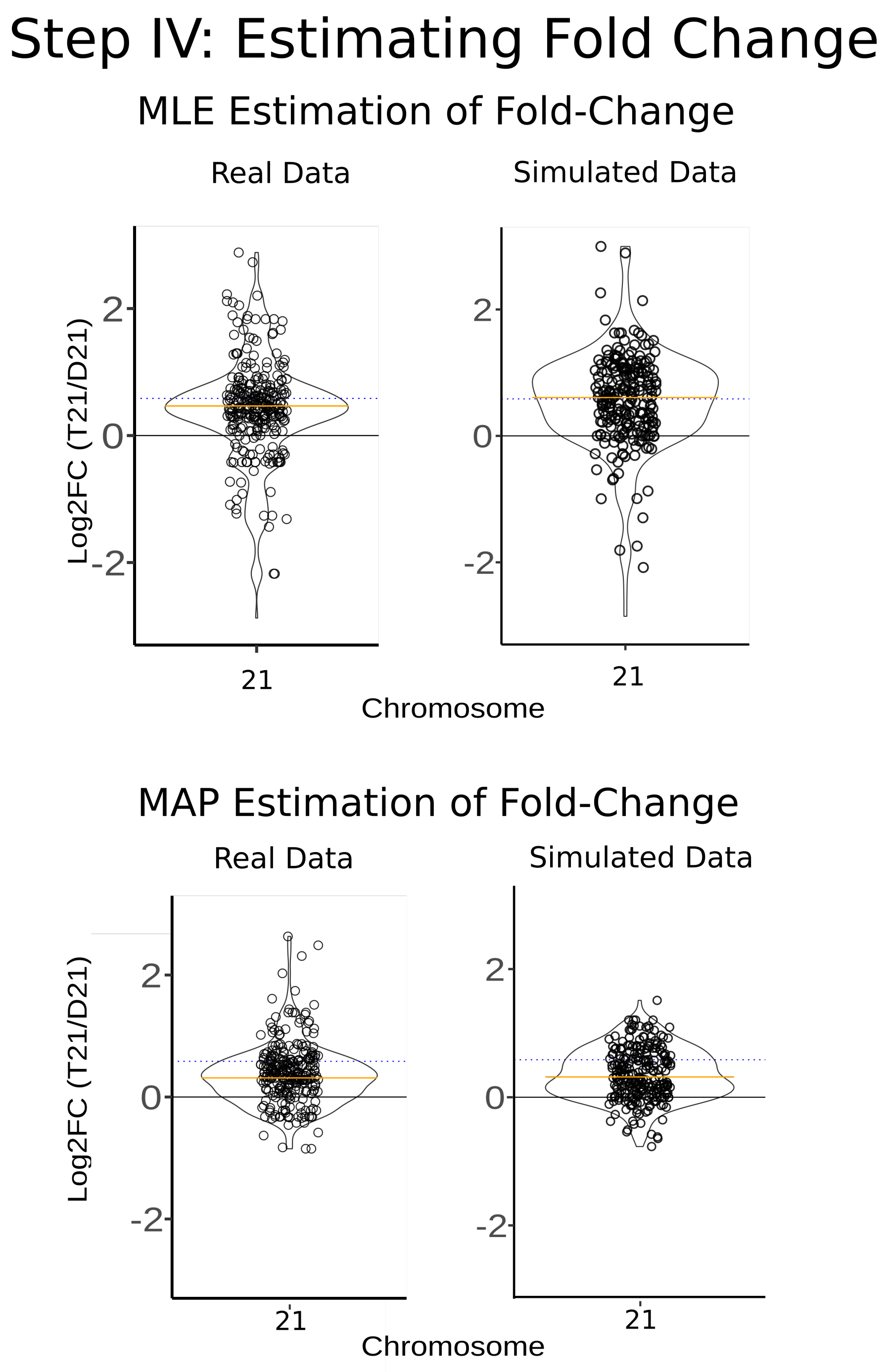

Fig S 15: **Violin plots of the fold change of chromosome 21 genes in real RNA-seq and simulated data, using maximum a posteriori estimates of fold change.** Due to fold change shrinkage, median fold change estimates are further pushed towards 0 with MAP estimation. Median fold change for each plot indicated with an orange line (MLE: RNA-seq MFC: 1.41, Simulated data MFC: 1.52; MAP: RNA-seq MFC: 1.24, Simulated data MFC: 1.21).

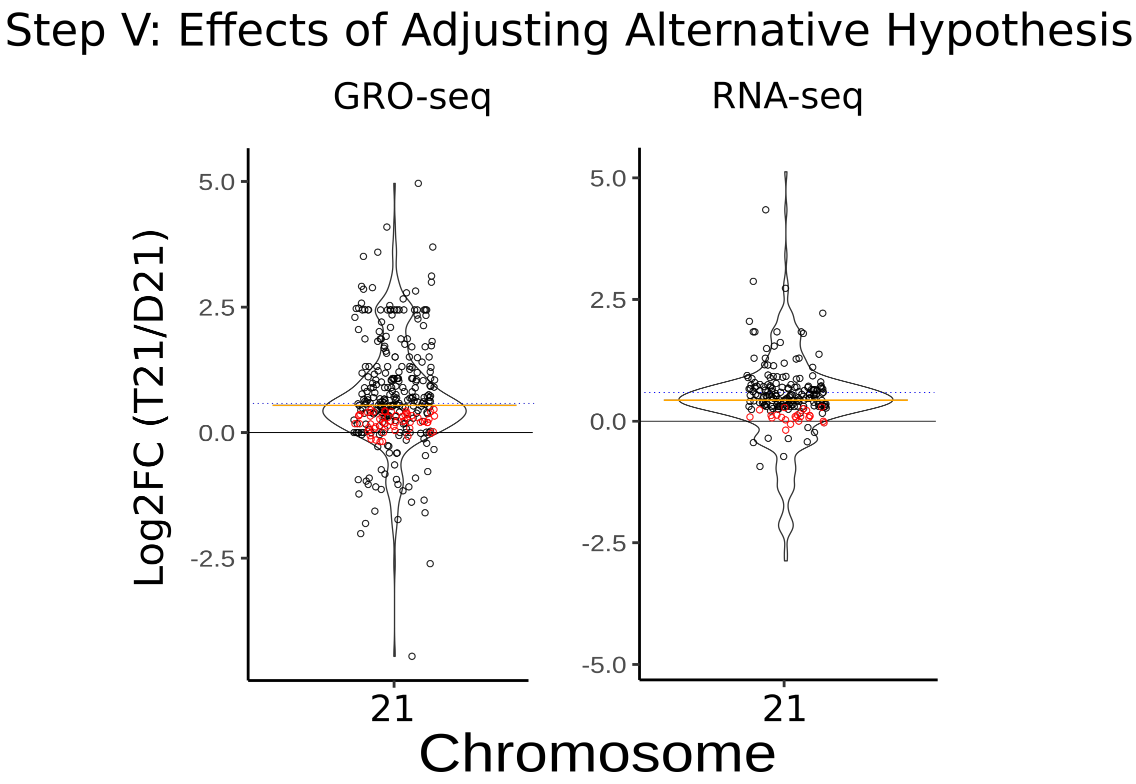

Fig S 16: **Differential analysis with adjusted alternative hypothesis (|LFC| < log2(1.5)).** Significant genes (red) are those which are significantly below the expected value of 1.5 (dotted blue line). Median fold change for each plot indicated with an orange line. Left: GRO-seq, 56 significant genes. Right: RNA-seq, 20 significant genes

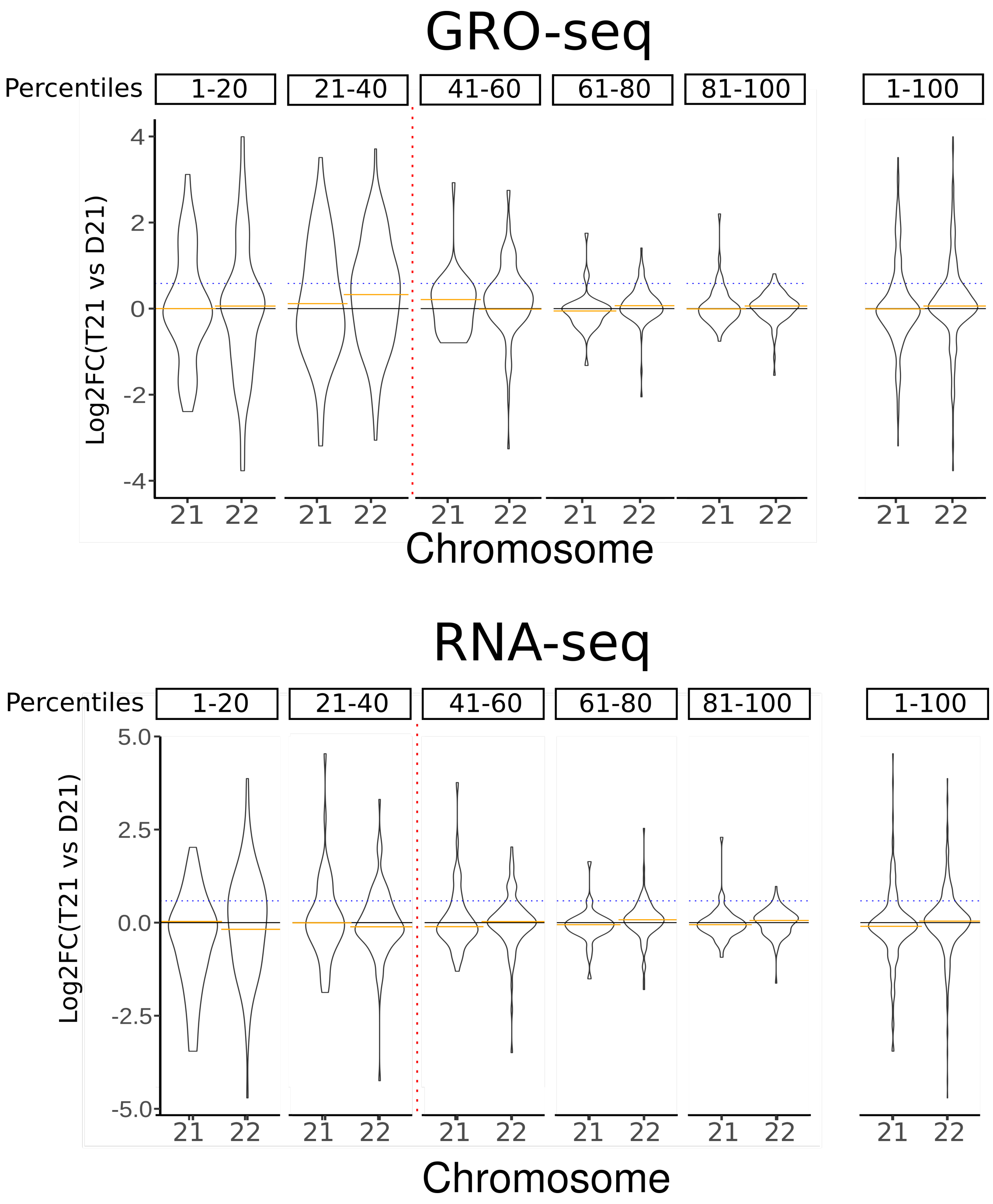

Fig S 17: **Fold change estimations (T21 vs D21) across expression levels in GRO-seq (top) and RNA-seq (bottom), after using a trisomy-aware pipeline.** Median fold change for each group indicated by orange line. Genes which fell below the red dotted line (percentiles to the left of the dotted line) are considered lowly expressed and subject to high variance, and are thus excluded from downstream analysis. See also Supplemental Fig 8 for fold change estimations without a trisomy-aware pipeline.

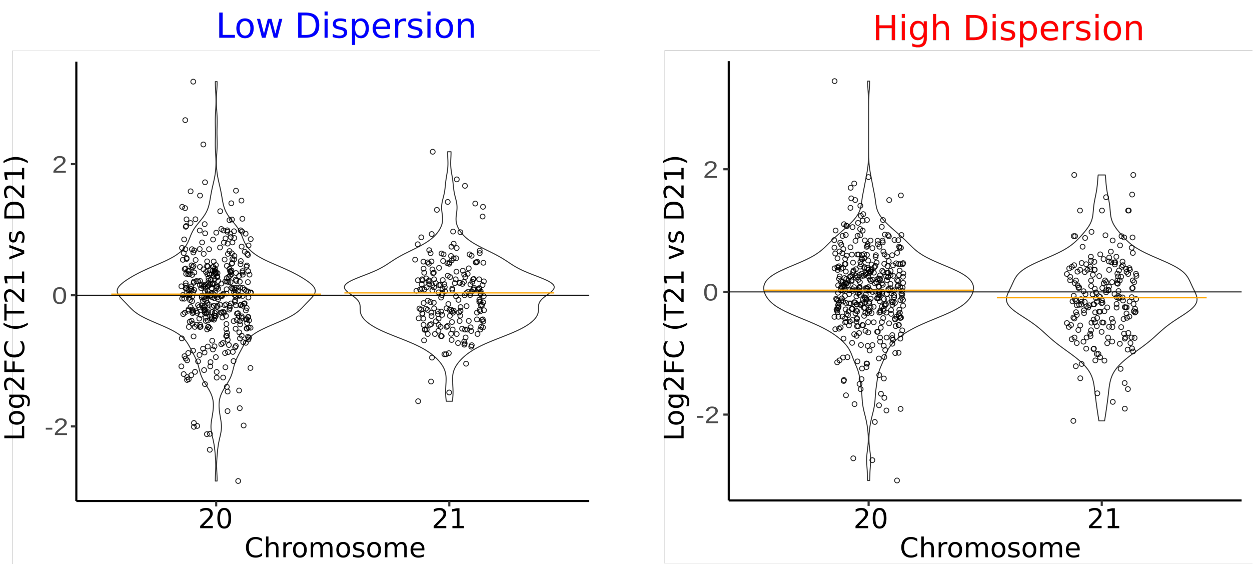

Fig S 18: **Fold-change estimates of simulated T21 and D21 data sets, using low and high dispersion estimates.** Fold-change estimates were adjusted to account for trisomy, as in Fig 2G. Low dispersion: a=.01, b=1. High dispersion: a=.05, b=30. Median fold changes indicated by orange lines.

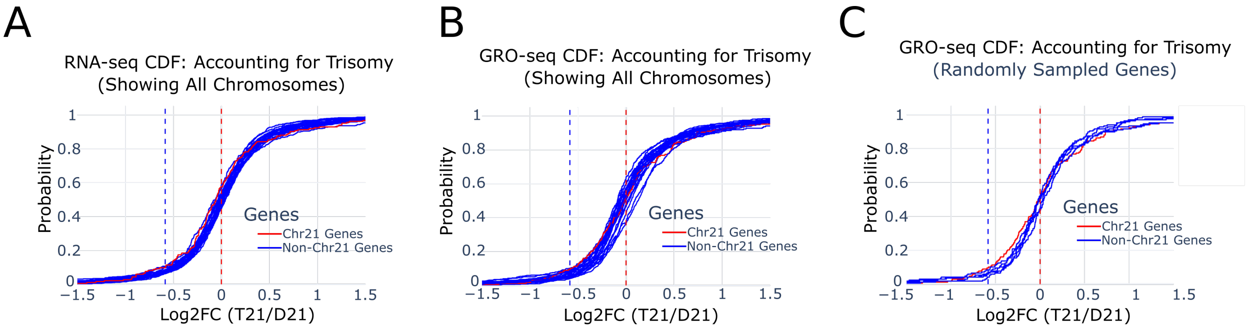

Fig S 19: **Cumulative distribution plots of fold changes in trisomy aware RNA-seq and GRO-seq analyses.** Vertical dotted red line indicates a log2 fold change of 0. Vertical dotted blue line indicates a log2 Fold change of -log2(1.5). (A) CDF of RNA-seq fold changes on all chromosomes (blue solid lines) with chromosome 21 indicated in red. (B) Same as (A), but using GRO-seq data. (C) CDF of GRO-seq fold changes using chromosome 21 genes (red) and a random subsample of an equivalent number of non-chromosome 21 genes (blue) which normalizes for size of chromosome.

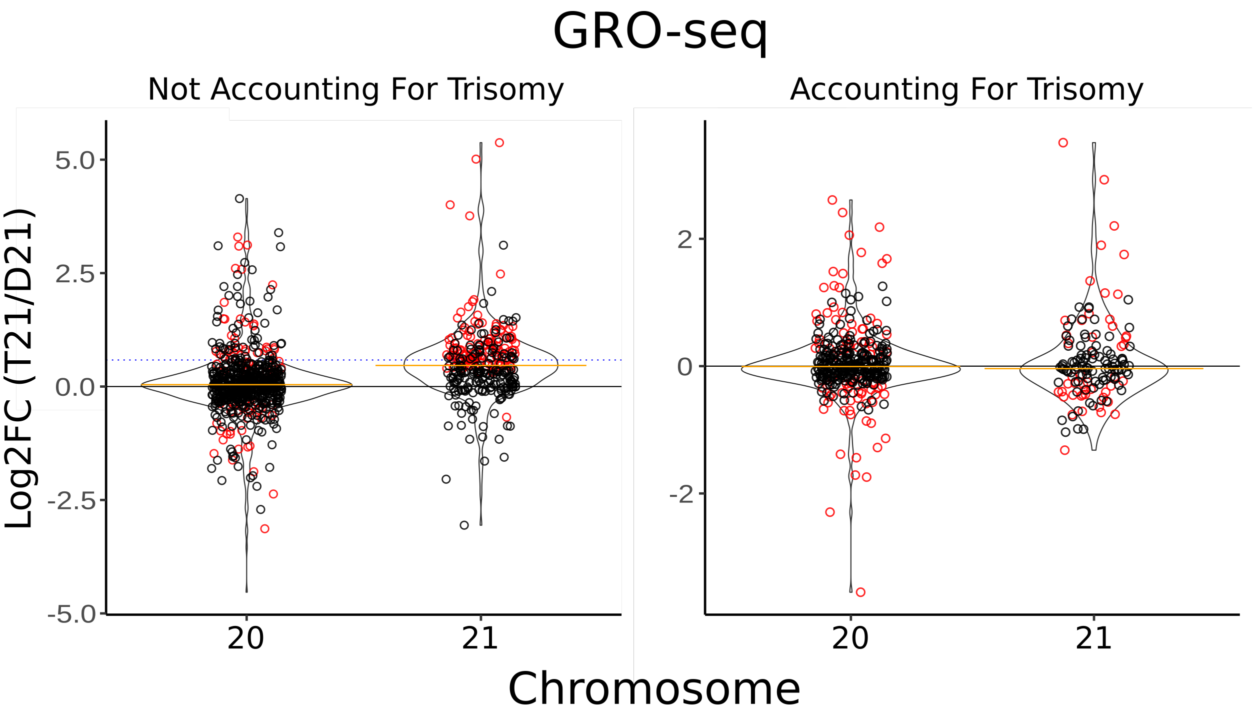

Fig S 20: **Violin plots depicting fold change estimates for chromosome 20 and 21 genes between T21 and D21 GRO-seq datasets.** (Left) Default analysis not accounting for the ploidy of the samples. (Right) Adjusted analysis correcting for ploidy differences between the samples. Median fold changes indicated by orange lines.

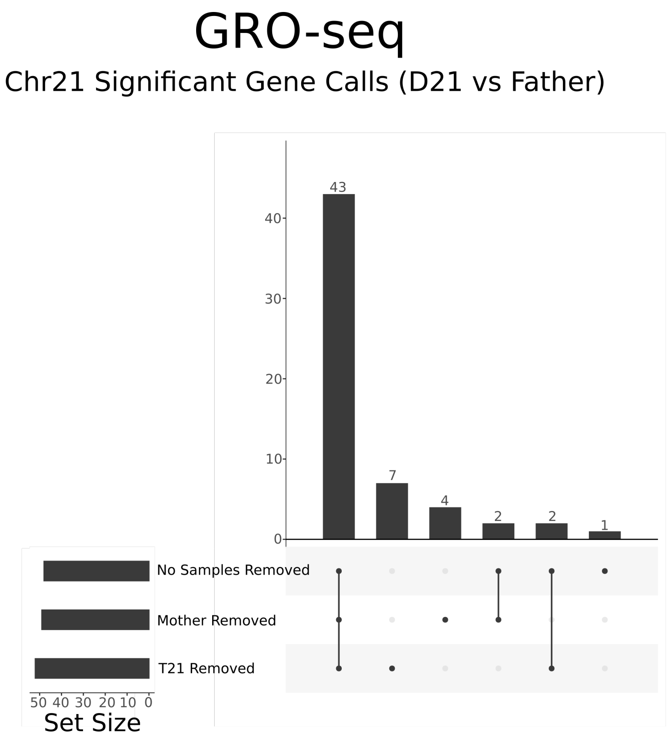

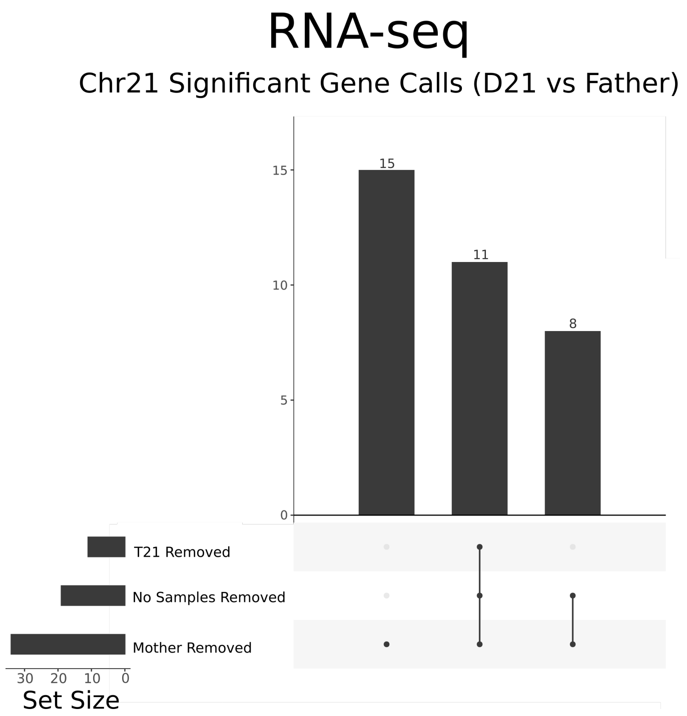

Fig S 21: **UpSet plots indicating overlap of significant gene calls on chromosome 21 comparing two disomic individuals.** We compare the D21 brother to the father and identify chromosome 21 encoded statistically significant genes (padj<.01). Three cases shown: we normalize to the entire family (no samples removed), remove the T21 sample or the mother sample is removed (as a control).

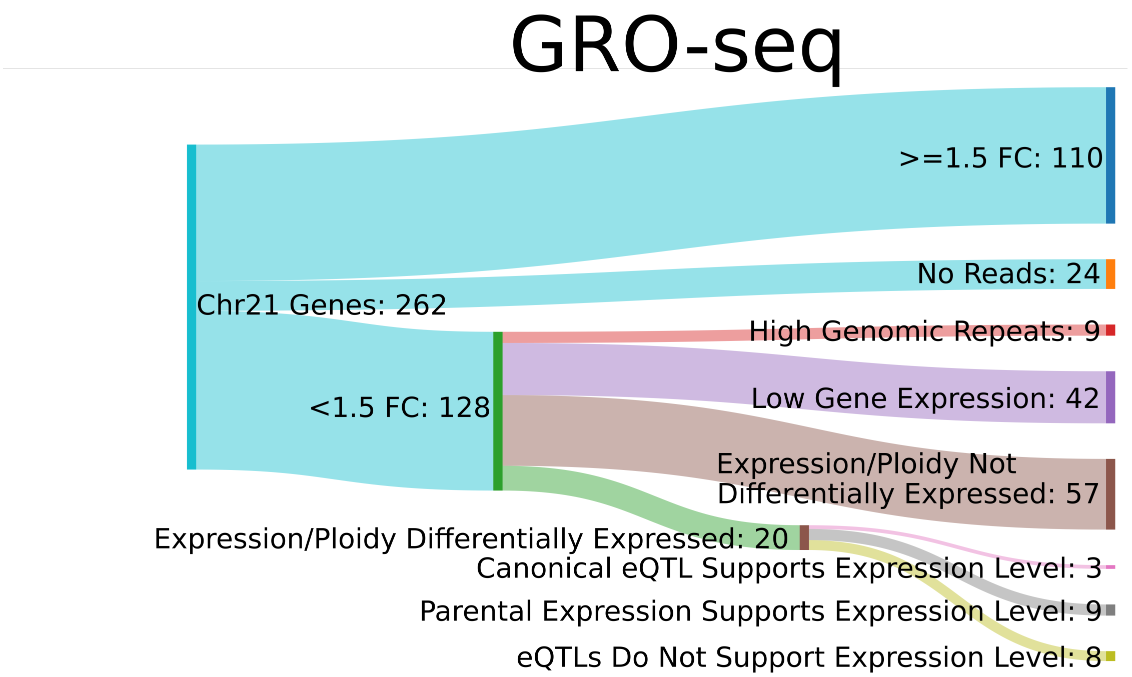

Fig S 22: **Sankey diagram indicating explanations of fold changes for chromosome 21 genes in GRO-seq (for RNA-seq, see Fig. 3D**. Nearly all genes with fold change lower than 1.5 can be explained by technical factors, leaving only 8 genes whose expression is below expectation that are not accounted for by genetic variation.

Supplementary References

[1] R. Bi and P. Liu. Sample size calculation while controlling false discovery rate for differential expression analysis with rna-sequencing experiments. *BMC Bioinformatics*, 17(1):146, Mar 2016.
